# Alanine Zipper-Like Coiled-Coil Domains Are Necessary for Homotypic Dimerization of Plant GAGA-Factors in the Nucleus and Nucleolus

**DOI:** 10.1371/journal.pone.0016070

**Published:** 2011-02-10

**Authors:** Dierk Wanke, Mareike L. Hohenstatt, Marek Dynowski, Ulrich Bloss, Andreas Hecker, Kirstin Elgass, Sabine Hummel, Achim Hahn, Katharina Caesar, Frank Schleifenbaum, Klaus Harter, Kenneth W. Berendzen

**Affiliations:** Center of Plant Molecular Biology (ZMBP) Plant Physiology, University of Tübingen, Tübingen, Germany; University of South Florida College of Medicine, United States of America

## Abstract

GAGA-motif binding proteins control transcriptional activation or repression of homeotic genes. Interestingly, there are no sequence similarities between animal and plant proteins. Plant BBR/BPC-proteins can be classified into two distinct groups: Previous studies have elaborated on group I members only and so little is known about group II proteins. Here, we focused on the initial characterization of AtBPC6, a group II protein from Arabidopsis thaliana. Comparison of orthologous BBR/BPC sequences disclosed two conserved signatures besides the DNA binding domain. A first peptide signature is essential and sufficient to target AtBPC6-GFP to the nucleus and nucleolus. A second domain is predicted to form a zipper-like coiled-coil structure. This novel type of domain is similar to Leucine zippers, but contains invariant alanine residues with a heptad spacing of 7 amino acids. By yeast-2-hybrid and BiFC-assays we could show that this Alanine zipper domain is essential for homotypic dimerization of group II proteins *in vivo*. Interhelical salt bridges and charge-stabilized hydrogen bonds between acidic and basic residues of the two monomers are predicted to form an interaction domain, which does not follow the classical knobs-into-holes zipper model. FRET-FLIM analysis of GFP/RFP-hybrid fusion proteins validates the formation of parallel dimers *in planta*. Sequence comparison uncovered that this type of domain is not restricted to BBR/BPC proteins, but is found in all kingdoms.

## Introduction

Eukaryotic gene expression is tightly controlled by enhancer and silencer elements. Additionally, in between these DNA regions, insulator elements have been identified in animals, which are bound by proteins, that mediate insulator function and prevent illegitimate activation or repression of neighboring loci [1]. In *Drosophila melanogaster* about twenty years ago the GAGA-factor (GAF) encoded by the *Trithorax-like* (*Trl*) gene was identified to bind to GAGA DNA-motifs inside such insulator regions [2], [3]. Ten years later, a second *Drosophila* protein, Pipsqueak (Psq), was identified and shown to bind to the same GAGA-motif as GAF, but with a structurally unrelated DNA-binding domain [4], [5]. Although GAGA-motif binding proteins have also been described in other species like plants and humans [5], [6], almost all the information on these proteins and their functions comes from *Drosophila*.

More recent studies revealed that GAGA-binding proteins aggregate into higher order complexes that locally replace nucleosomes to form a specific chromatin environment [7], [8]. However, functions of GAGA-motif binding proteins are more diverse and can also be linked to epigenetic regulation of homeotic genes e.g. by recruiting silencing factors to specific sites, as well as influencing the promoter-proximal pausing of RNA Polymerase II (Pol II) [5], [9], [10], [11].

In plants, GAGA-motif binding transcription factors had first been identified in soybean via their ability to bind to the (GA/TC)n - dinucleotide repeat enhancer element of the *GSA1*-promoter [6].

In barley, the Barley B Recombinant (BBR) protein was the first functionally characterized GAGA-motif binding transcription factor, which was shown to be an essential regulator of the homeobox gene *Barley Knotted 3* (*BKn3*), the ortholog of the well known homeobox gene *Knotted 1* (*Kn1*) from *Zea mays* [12], [13], [14], [15]. In the dominant-active *Hooded* (*K*) phenotype of barley, a homeotic transformation of the floral organs had been observed due to the constitutive ectopic *BKn3* expression in all floral organs [13], [16].

The mutation of the *Hooded* phenotype was mapped to an intragenic duplication of a 305 bp region inside the fourth intron of *BKn3*, which containes a (GA/TC)_8_- dinucleotide repeat motif [13], [16]. The BBR protein was identified through its specific binding to this (GA/TC)_8_- dinucleotide repeat enhancer element [13]. The ectopic expression of BBR in *Nicotiana tabaccum* results in an enlargement of all plant organs [13].

A BBR member from Arabidopsis named Basic Pentacysteine (BPC) protein, was found essential for the activation of INNER NO OUTER (INO) by binding to a GA/TC-dinucleotide rich sequence its promoter [17]. Basic pentacysteine refers to five highly conserved Cysteine residues in the basic zinc finger-like DNA-binding domain, the characteristic hallmark of the plant specific BBR/BPC protein family [17], [18].

BBR and BPC proteins are nuclear targeted proteins with a plant specific zinc finger like DNA-binding domain at their carboxy termini [13], [17].

Moreover, cooperative binding of BPC1 proteins to GA-rich motifs in the SEEDSTICK (STK) promoter region leads to a condensation and looping of DNA [18], similar to what has been described for GAGA-motif binding factors from *Drosophila* [19], [20].

Sequence comparison revealed that at least two major groups of BBR/BPC proteins can be differentiated: group I and group II [17]. Previous publications have concentrated on the functions of group I proteins, and as such, there is no information on group II BBR/BPC proteins.

In this study we analyzed two phylogenetically conserved domains of *Arabidopsis* AtBPC6 as a first step to understand the function of group II BBR/BPC proteins in plant cells. First, a 31 amino-acid long region was found to be essential and sufficient for the localization of AtBPC6 to the nucleus and the nucleolus. Second, a novel type of a zipper-like coiled-coil was found to functions as dimerization domain, and this novel coiled-coil domain is present in all kingdoms, from bacteria to plants and humans. We show that *Arabidopsis* group II BBR/BPC proteins can form homotypic dimers in parallel alignment via this coiled-coil domain in yeast and in plant cells.

## Results and Discussion

### *Drosophila* and Plant GAGA-binding proteins are phylogenetically unrelated

**Figure 1:**
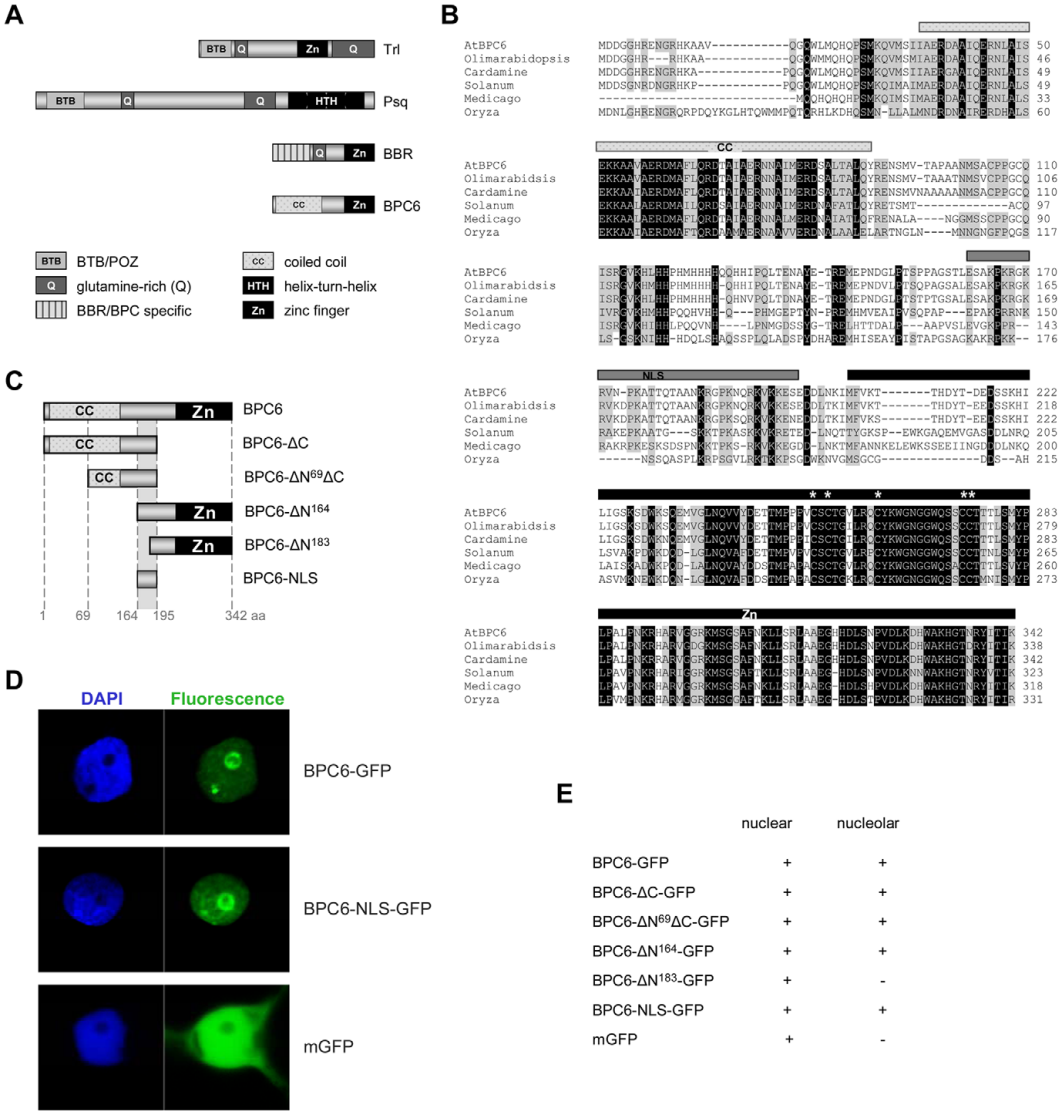
Protein organization and subcellular localization of GAGA-motif binding proteins. (**A**) Schematic representation of *Drosophila* and plant GAGA-motif binding proteins. Locations of homologous domains are shown for the DNA-binding domains (black), putative protein interaction domains (grey) and Q-rich domains (dark grey). (**B**) Protein sequence alignment of *At*BPC6 orthologs. Conserved amino-acids are highlighted, invariant positions (black) and positions that are preserved in at least half of the aligned sequences (grey). Sequences are retrieved from Olimarabidopsis (*Olimarabidopsis pumila*), Cardamine (*Cardamine pratensis*), Solanum (*Solanum lycopersicum*), Medicago (*Medicago truncatula*) and Oryza (*Oryza sativa*). Three distinct domains with putative conserved functions are predicted and highlighted by bars on top of the sequences: Coiled-coil (checked-grey), nuclear localization signal (dark grey) and zinc-finger like DNA-binding domains (black). Invariant Cystein positions within the basic DNA-binding domain are indicated by asterisks. (**C**) Schematic representation of AtBPC6 fragments used for functional analyses and hybrid protein fusions. (**D**) Laser confocal microscopy analysis of BPC6-GFP, BPC6-NLS-GFP and mGFP localization in *Nicotiana benthamiana* epidermis cell nuclei. (**E**) Hetreologous expression in *Nicotiana benthamiana* epidermis cells identified the 31 amino-acid long NLS to be responsible for targeting GFP-fusion proteins to the nucleus and the nucleolus.

The BBR/BPC proteins of plants do not share sequence similarities with the *Drosophila* GAGA-motif binding proteins Trl and Psq (Figure 1A), although all of them bind to purine rich GA-dinucleotide repeat motifs. Trl and Psq share the BTB/POZ-domains as protein interaction surfaces and glutamic acid (Q) rich pattern [5]. However, both proteins differ in their DNA-binding domains. While Psq possesses a Helix-Turn-Helix domain for DNA-binding at its C-terminus, Trl binds GAGA-DNA motifs by a zinc-finger like domain. Similar to Trl, plant specific BBR/BPC proteins have a highly conserved zinc-finger like DNA-binding domain. Trl and Psq mediate partially redundant and opposing functionality [4], [10], [11], [21]. One might propose that the two major groups of BBR/BPC proteins also mediate different functions by analogy. Based on protein sequence analysis, group I proteins share at least three distinct domains (Figure 1A): a BBR/BPC specific domain of unknown function at the N-terminus, a central nuclear localization signature and the conserved DNA-binding domain at the C-terminus. Similar to Trl or Psq, some group I members, such as the barley BBR, contain glutamic acid (Q) or histidin-glutamic acid (HQ) rich patterns [13]. Both motifs are known to activate gene expression in plants and other eukaryotes [22], [23], [24], [25], [26]. For group II proteins, such as AtBPC6 from *Arabidopsis*, only two domains were predictable from sequence analyses, which are a coiled-coil signature and the conserved DNA-binding domain shared with group I members.

### Alignment of group II BBR/BPC-proteins

To gain an insight into possible domains present in group II BBR/BPC proteins, we identified AtBPC6 orthologs from two close relatives of *Arabidopsis*, *Olimarabidopsis pumila* and *Cardamine pratensis*. Protein sequences of more distantly related group II proteins from rice, tomato and *Medicago truncatula* were retrieved from GenBank. Sequence comparison revealed several regions that exhibited local conservation in their amino acid positions (Figure 1B). Besides the highly conserved DNA-binding domain at the C-terminus and the predicted coiled-coil domain at the N-terminus, two evolutionary conserved regions between AtBPC6 orthologs were discovered: first, a region spanning amino acid residues 110–152 that has no homology to any previously described structure. Second, residues 164–195 are rich in basic amino acids and its sequence is similar to nuclear localization signatures [27], [28], [29], [30]. Indeed, a monopartide NLS motif (^192^KxKK^195^) is predicted by analysis algorithms. However, a second basic motif ^184^KRxxK^188^, which resembles NLS motif consensi, is positioned only 4 amino acids apart from the first; and a third NLS-like motif (^165^KPKRxKR^171^) is phylogenticaly conserved, which has not been recognized by any prediction algorithms.

### AtBPC6 localizes to the nucleus and the nucleolus

The sequence alignment of AtBPC6 orthologs and structural prediction programs (Supporting Figure S1) led to clues where to dissect the protein into fragments for further functional analyses (Figure 1C). The full-length cDNA and five fragments of AtBPC6 were cloned in ENTRY-vectors and recombined into binary expression vectors to make GFP-fusion proteins. We identified the residues 164–195 to be essential and sufficient for the targeting of AtBPC6 protein to the nucleus and the nucleolus in transient expression assays using *Agrobacterium* mediated transformation of *Nicotiana benthamiana* epidermis cells (Figure 1D).

All fusion proteins containing this 31 amino acid long peptide signature exhibited the same localization as the full-length AtBPC6-GFP (Figure 1E). In the nucleoplasm the GFP-fusion proteins co-localized with DNA or in distinct speckle-like domains. At the nucleolus AtBPC6-GFP fluorescence signals formed a ring-shaped localization pattern around the central nucleolar cavity (Supporting Figure S2).

Interestingly, BPC6-ΔN^183^-GFP also localized to the nucleus, but not to the nucleolus (Supporting Figure S3). Although the predicted monopartide NLS motif (^192^KxKK^195^) and the second basic motif ^183^KRxxK^187^ were contained within the BPC6-ΔN^183^-GFP protein, it was unable to localize to nucleolus. This suggests that all three NLS-like motifs are required for recognition and the proper targeting of AtBPC6 to the nucleolus.

### AtBPC6 is retained in isolated nuclei

**Figure 2:**
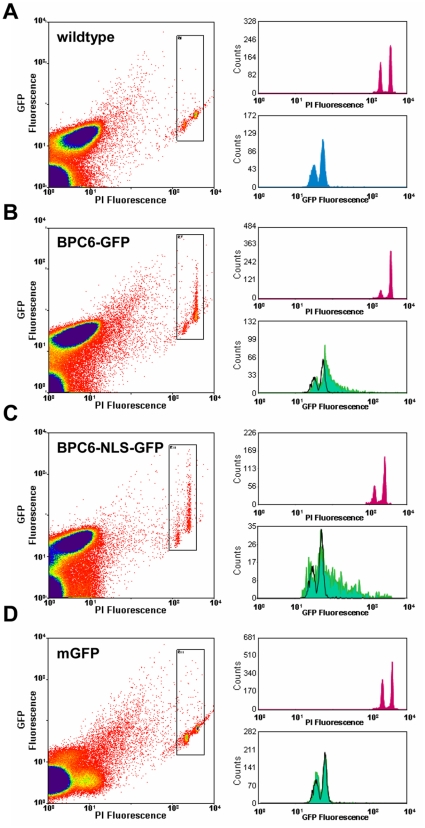
Stable localization of BPC6 fusion proteins to the nucleus. Quantification of fluorescence signals in *Nicotiana benthamiana* nuclei using flow-cytometry. Nuclei are prepared from non-transformed wildtype cells (**A**) and epidermis cells expressing BPC6-GFP (**B**), BPC6-NLS-GFP (**C**), mGFP (**D**). GFP fluorescence and DNA content is measured after incubation of nuclei in propidium iodide (PI) solution. Representative signal intensity plots of PI- and GFP-fluorescence for ∼250000 events are shown (left). Histograms of PI-fluorescence (top right) and GFP-fluorescence (bottom right) intensity counts are given for each of the measurements. For comparison, normalized background fluorescence of non-transformed wildtype cells are accompanying histograms of GFP-transformed cells (black lines). BPC6-GFP and BPC6-NLS-GFP are retained inside the nucleus in a stabile manner, while mGFP leaks out and gives background signals perfectly overlapping with wildtype (black line). Black arrowheads indicate significant retention of the GFP signals inside the nuclei.

The import into the nucleus across the membranes of the nuclear envelope is governed by importins localized at the nuclear pores that recognize the NLS signatures of the proteins and actively recruit them for translocation [27], [30]. However, the nucleolus is not surrounded by an additional membrane and the localization to this subnuclear domain likely requires interaction with other components in the nucleus or the nucleolus [31], [32], [33]. To test whether AtBPC6 retained in the nucleus, we performed a flow cytometric analysis on isolated *Nicotiana benthamiana* nuclei expressing BPC6-GFP and BPC6-NLS-GFP compared to free mGFP. A propidium iodide (PI) co-staining was performed to restrict the analysis to identify released nuclei (Figure 2). We showed previously that mGFP localized to the cytoplasm and inside the nucleus (Figure 1D and Supporting Figure S3), as it is a small protein that does not necessarily need an active transport by the nuclear import machinery. Thus, we expected a leaking of mGFP from the nucleus during the isolation and incubation procedures. Indeed, the flow cytometric fluorescence measurements of mGFP expressing cells exhibited no differences from untransformed wildtype nuclei (Figure 2D).

In contrast, BPC6-GFP and BPC6-NLS-GFP expressing cells displayed a stabile GFP fluorescence inside the nuclei (Figures 2B and C). Both fusion proteins were prevented from being washed out during isolation and incubation, which implies an active retention due to interaction with components present inside the nucleus. Hence, the 31 amino acids of the NLS were necessary and sufficient for localization and nuclear retention.

### Dimerization studies with Yeast-two-Hybrid Assay

It is known for the Drosophila GAGA-motif binding proteins that dimerization is a prerequisite for their function [5], [34], [35]. Moreover, interaction and cooperative binding to DNA has already been inferred for AtBPC1 on the basis of DNA-bending capabilities *in vitro* [18]. Therefore, we analyzed the ability of AtBPC6 to form dimers *in vivo* that might also give us a clue to the nuclear/nucleolar retention of the proteins during the flow cytometry. We exploited the yeast-2-hybrid assay to systematically test BPC6 prey fragments against different BPC bait proteins.

**Figure 3:**
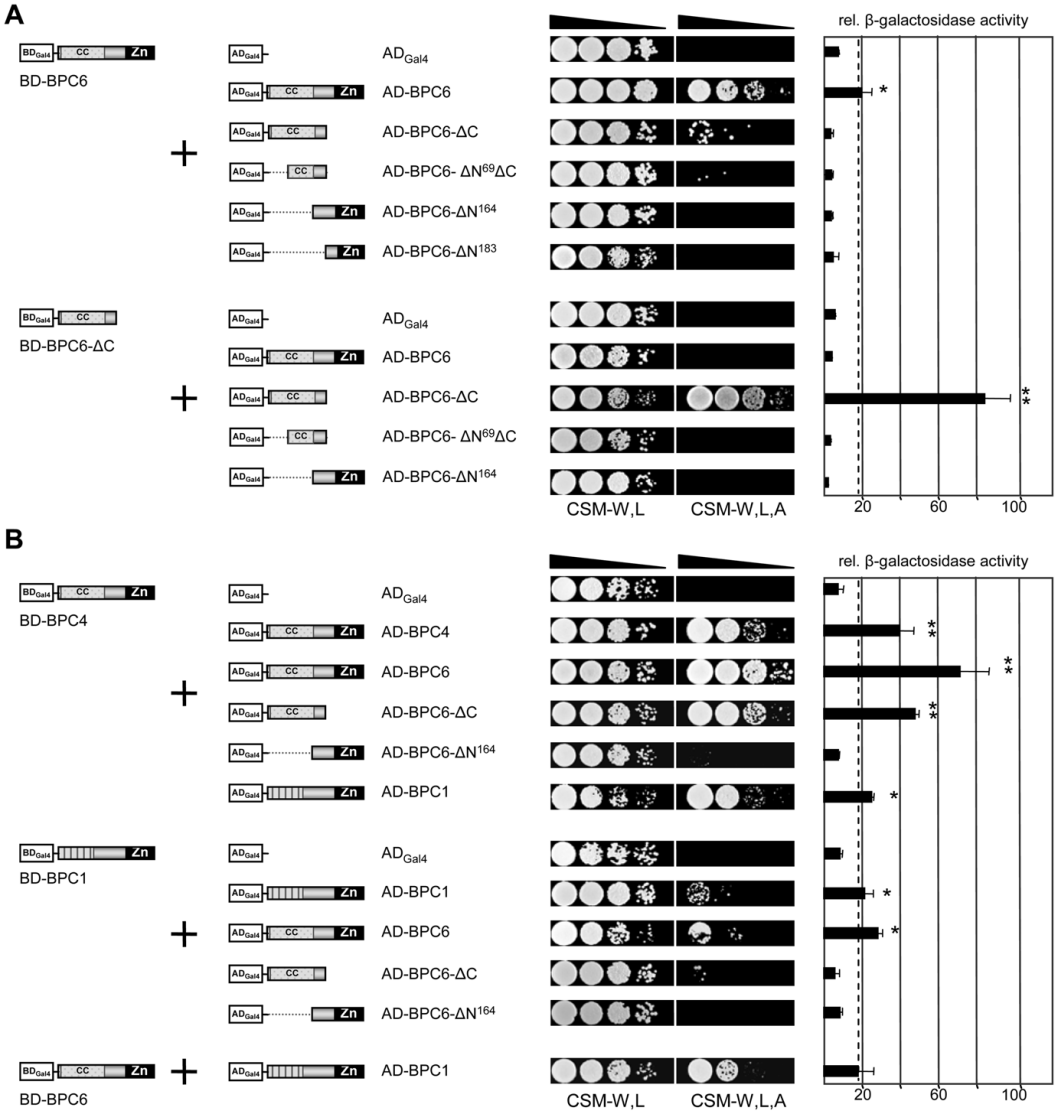
Coiled coil domains of Arabidopsis group II BBR/BPC proteins are essential for homotypic dimerization. Interaction analyses with hybrid fusion proteins of AtBPC6 and its fragments by yeast two-hybrid are shown (**A**). The BPC6-ΔC fragment containing the coiled-coil domain interacts with full-length BPC6 and BPC6-ΔC in a hybrid-fusion dependent manner. Interaction of group II with group I and II proteins in the yeast two-hybrid system is tested with AtBPC1, AtBPC4 and AtBPC6 hybrid-fusion proteins (**B**). The coiled-coil domains of group II hybrid proteins BPC4 and BPC6 are essential for homotypic dimer formation. Interaction is tested by growth on adenine-deficient CSM selection media and β-galactosidase reporter activity. Enzymatic activity of the β-galactosidase reporter is quantified from triple measurements of MUG substrate assays and three independent transformations [n = 9]. Statistical background for no significant interaction is calculated from combinations with empty AD-clones and is shown as dotted line. Asterisks mark combinations of hybrid fusion proteins that mediate significantly increased reporter enzyme activities over the background: (**) indicates strong and highly significant (p≤0,0001) interaction; Weak but significant interaction (p≤0,01) is indicated by (*).

Indeed, AD-BPC6 and BD-BPC6 expressing yeast grew on selective media and displayed significant β-galactosidase activity, which is indicative for the formation of AtBPC6 homodimers (Figure 3A). After testing all five fragments and the full-length BPC6 protein in all possible AD- and BD-combinations (data not shown), we found that the fragment BPC6-ΔC is responsible for the homodimerization of the AtBPC6 protein (Figure 3A). The BPC6-ΔC fragment contains the entire N-terminus harboring the conserved coiled-coil domain (Figure 1 and Supporting Figure S4). A fragment that lacks the C-terminus and half of the coiled-coil region, BPC6-ΔN^69^ΔC, could not evoke reporter gene activity or confer growth on selective media (Figure 3A), which indicates that the entire coiled-coil region is required for dimerization. Interestingly, both AD- or BD-combinations of BPC6-ΔC with the full-length BPC6 protein did not exhibit any significant reporter activities, which suggest a negative regulatory function on the dimerization by one of the other AtBPC6 domains.

To test the ability of AtBPC6 to form heterodimers with other BPC proteins, we tested AtBPC4, another group II protein in Arabidopsis, and AtBPC1, a group I member that has been studied previously [18].

AtBPC4 also contains the coiled-coil domain characteristic for group II proteins, and indeed, we found AD-BPC4 able to form homodimers with itself (Figure 3B). Moreover, BPC4 combinations with either BPC6-ΔC or BPC6 conferred growth on selective media and exhibited significant β-galactosidase activity, which both indicates homotypic dimer formation between group II proteins in the yeast cells.

Here again, dimerization could only be detected with constructs that express the entire coiled-coil domain.

However, strong yeast growth and reporter gene activity could be detected with full-length AD-BPC4 protein and BD-BPC6-ΔC. Hence, we have to conclude that AtBPC4 lacks the inhibitory function that acts negatively on BPC6-ΔC and its homodimerization with full-length BPC6. We can not rule out that this essential function is contained in the most N-terminal part of AtBPC6 and AtBPC4 that shares much less similarities than the coiled-coil regions of both proteins, which are highly conserved.

When testing the group I protein AtBPC1 for homo- and heterodimerization, a weak growth on selective media was detected accompanied by β-galactosidase activity (Figure 3B).

This supported the idea of homo- and heterotypic dimer formation within and between the different groups of BBR/BPC proteins in the heterologous yeast expression system. However, the homotypic interaction between the group II proteins that was mediated by the characteristic coiled-coil domain was the strongest in all combinations. Moreover, the retention of BPC6-NLS inside the nucleus and nucleolus can possibly not be explained by interaction with BBR/BPC family members, as none of its tested fragment combinations mediated reporter activity. Hence, other jet unknown interaction partners will likely be responsible for this localization.

However, we consider an additional observation as noteworthy: None of the BPC bait constructs evoked any transactivation of the yeast reporters (Figure 3). This implies that Arabidopsis group II BPC proteins as well as AtBPC1 lack eukaryote transcription activation domains.

### BiFC supports the formation of BPC homo- and heterodimers *in planta*

**Figure 4:**
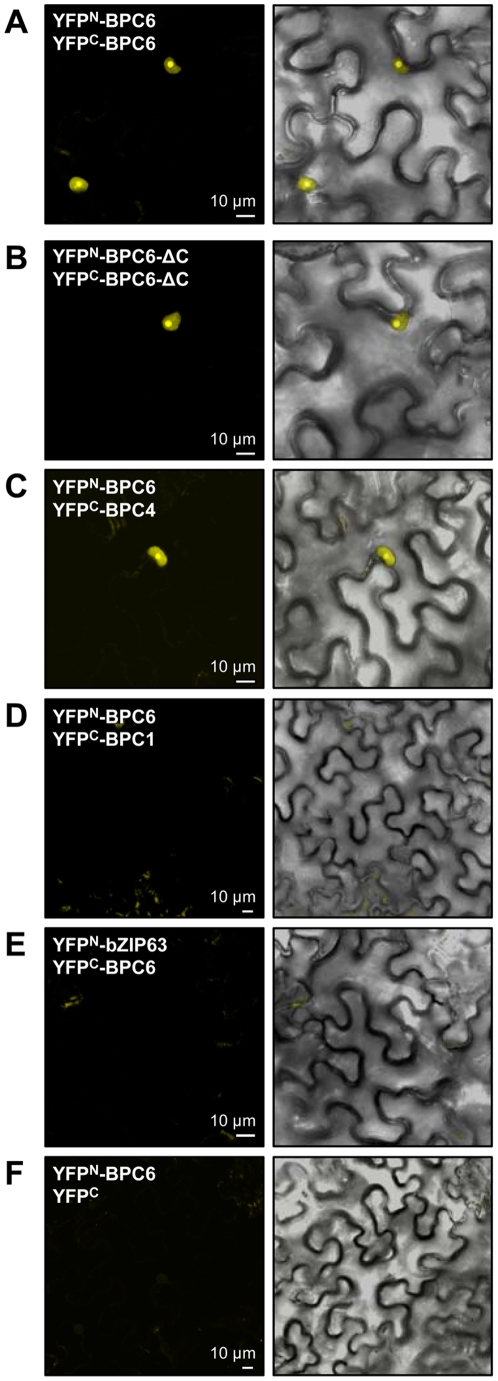
Arabidopsis group II proteins BPC4 and BPC6 form homotypic dimers *in planta*. Bi-molecular fluorescence complementation (BiFC) is used for *in planta* interaction studies. (**A–F**) Expression of indicated BPC1, BPC4 and BPC6 split-YFP hybrid fusion proteins are examined in transiently transformed *Nicotiana benthamiana* epidermal cells. The coiled-coil domain is essential for homotypic dimerization of BPC4 and BPC6 in the nucleus and the nucleolus.

To test whether the interactions found in the heterologous yeast system can also be detected *in planta*, we made use of the bimolecular fluorescence complementation (BiFC) assay [36]. In transiently transformed *Nicotiana benthamiana* epidermis cells we could detect strong complemented YFP signals for homodimers of BPC6 and BPC6-ΔC as well as BPC6/BPC4 homotypic dimers (Figure 4). Thus, the interaction mediated by the coiled-coil domain takes place in the plant's nucleus and nucleolus. Consequently, both group II proteins BPC6 and BPC4 are localized to the same nuclear expression domains.

Heterotypic dimers of BPC1/BPC6 as was implicated by the yeast-two-hybrid assays could not be verified *in planta*, as the observed signals with this combination did not exceed the background fluorescence of the negative control combinations YFP^N^-BPC6/YFP^C^-bZIP63 or YFP^N^-BPC6/YFP^C^ (Figure 4). Therefore, an interaction between BPC1/BPC6 *in planta* is likely not existing under physiological conditions inside the plant's nucleus or it is very transient and, hence, below detection level.

### *In silico* Analysis of the novel Alanine Zipper-like Domain

**Figure 5:**
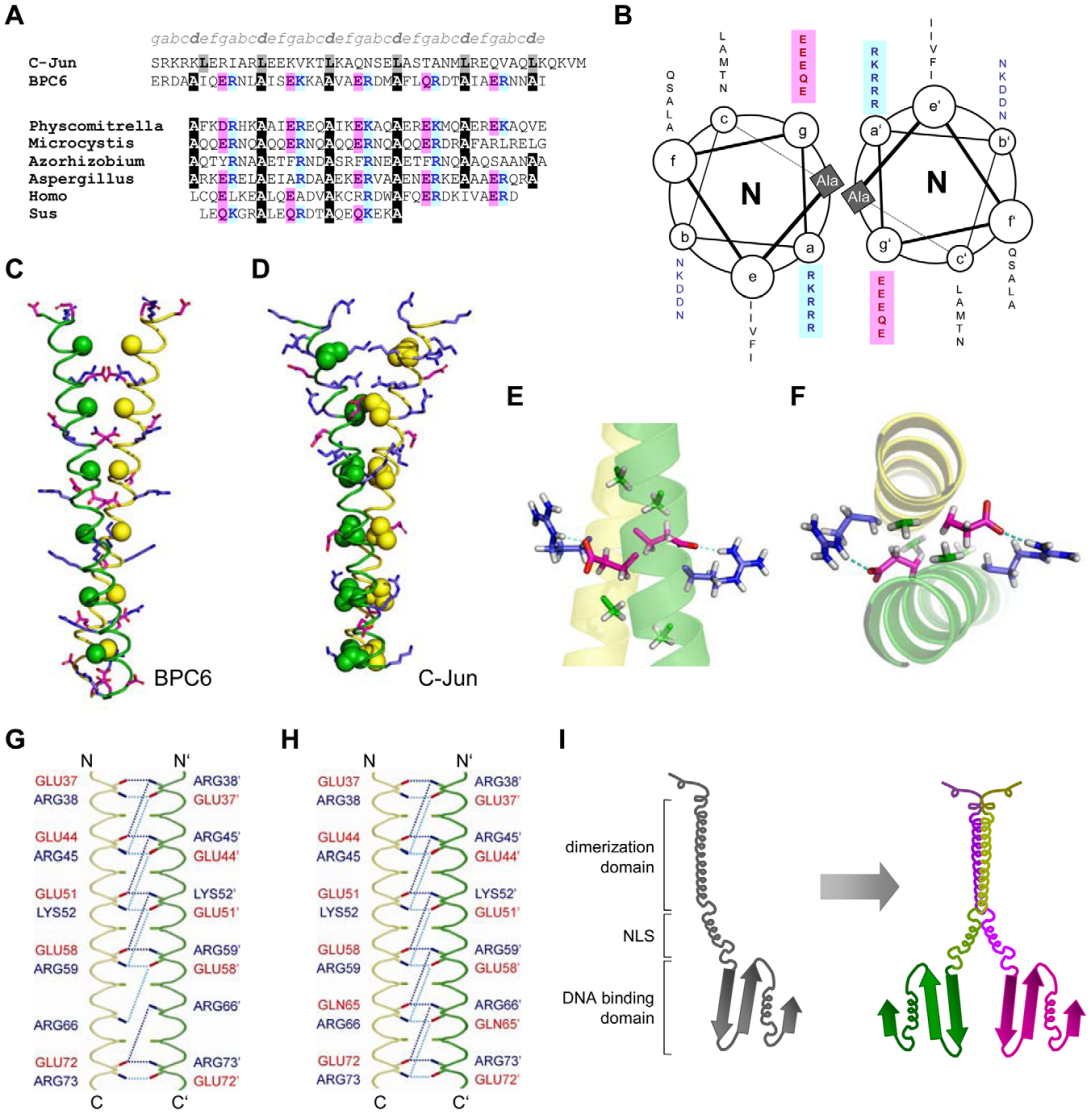
The Alanine zipper coiled-coil forms dimers via electrostatic interaction. (**A**) Alanine zipper regions of AtBPC6 and of other proteins are compared to the Leucine zipper of the C-Jun oncogene: *Physcomitrella*: [GenBank: AB292414], *Microcystis*: [GenBank: AP009552], *Azorhizobium*: [GenBank: ABA21837], *Aspergillus*: [GenBank: XM_750964], *Homo*: [GenBank: BC146794], *Sus*: [GenBank: XM_001925969]. Conserved Alanines at the ‘d’ positions of the Alanine zipper are highlighted with black background color. Conserved positions of positively or negatively charged residues are indicated in blue or in red colors, respectively. (**B**) Helical wheel diagram of an Alanine zipper homodimer. The diagram depicts the clockwise axial rotation of the helices as viewed from their N-termini. The conserved Alanines form a core and are flanked by the positive (blue) and negative (red) charged residues that oppose each other. The wheel starts with Ala^41^ and ends with Ala^76^. Ribbon dimer model of the AtBPC6 Alanine zipper (**C**) and the C-Jun Leucine zipper regions (**D**). Monomers are either shown in green or in yellow. Conserved alanines or leucines in the core of the respective dimer are displayed as ball structures. The Leucines of the C-Jun dimer are involved in binding and form a hydrophobic core, which cannot be seen in the AtBPC6 Alanine zipper. Enlarged side (**E**) and top (**F**) view of the Alanine zipper homology model. Positively (blue) and negatively (red) charged side chains embrace the alanines (green) and form salt bridges or hydrogen bonds (turquoise). Schematic overview of the residues that support the stable dimer structure of the homology modeled AtBPC6 Alanine zipper via salt bridges (**G**) or hydrogen bonds (**H**). Amino acids with positive or negative charges are given in blue or red, respectively. Schematic drawing of the BBR/BPC group II monomers and dimers (**I**). The so far characterized domains imply a functional model, in which the proteins form homotypic dimers within group II in a parallel manner. As a consequence, their two DNA-binding domains make contact to neighboring GAGA-motifs on the same side of the strand.

The occurrence of BBR/BPC-family proteins is restricted to the plant kingdom and its BPC-type DNA-binding domain is of monophyletic nature [17]. In contrast, the characteristic coiled-coil domain of group II BBR/BPC-proteins exhibits similarities to various proteins of all organism kingdoms, including brown algae, fungi as well as humans (Figure 5A and Supporting Figure S5). However, only few of these proteins have an assigned cellular role, e.g. *Hs*SMC1A (structural maintenance of chromosomes 1A), *Ss*MAP1A (microtubule-associated protein 1A) or *Sc*RPL7b (ribosomal protein L7) [37], [38], [39], however, some localize in the nucleus similar to BBR/BPC-proteins.

Characteristic of this BBR/BPC type coiled-coil domain is the presence of alanine residues with an even spacing of seven amino acids in the extended alpha-helical region (Figure 5A).

Similar domains have previously been described as Alanine zippers or Alacoils that form antiparallel coiled coils [40], [41], [42], [43], [44]. A distinguished structural feature are the alanines at either the ‘d’ position or the ‘a’ position of the helical register, which allows a very tight oligomeric (usually tetrameric) architecture of the coils [42], [44], [45]. Well studied examples are the antiparallel homodimeric coiled coils of the Lac repressor, the ROP protein or of tropomyosin [40], [41], [42], [43], [45], [46]. So far, only the tetrameric Alacoil of the HAMP domain in histidine kinases of the two-component system is known to assemble in parallel coils of four-helical bundles of homodimers [42], [47].

These reports suggested that group II BBR/BPC proteins do likely interact by the formation of antiparallel coils. In contrast, database searches revealed higher overall similarities with parallel pairing Leucine zippers: In Leucine zippers, the heptad spacing of aliphatic amino acid residues forms a hydrophobic inner core of the helical bundles, while hydrophilic residues stabilize the zipper region on the outside [45], [48], [49]. On the one hand, it has been reported that the side chain of alanines is too short to support a hydrophobic inner core of classical Leucine zipper dimers [49], [50], [51]. On the other hand, there are also evenly spaced aliphatic amino acids present in the BBR/BPC-type coils besides the highly conserved Alanines.

Hence, we were wondering whether the Alanine zipper-like motif is an unusual type structure that is still capable of forming parallel Leucine zipper-like coils instead of antiparallel helical bundles. To address that question, we used several publicly available prediction algorithms [52], [53], [54]. All of them predicted a coiled-coil region that does not form a Leucine zipper-like interaction domain, because of missing hydrophilic residues in the correct position of the alpha-helix (Figure 5A). We also tested the Alanine zipper motifs from other organisms (Figure 5A), but none of them was predicted to form Leucine zipper-like coils either (data not shown).

Besides the heptad positioned alanines (presumably at the ‘d’ position of the zipper), we found evenly spaced, negatively charged residues at the ‘g’-position of the helix accompanied by positively charged residues at the ‘a’-position (Figure 5A). To gain a better insight into the three dimensional positioning of the residues, the amino acids of the Alanine zipper were displayed in an alpha-helical wheel diagram (Figure 5B). Here, charged amino acids were directly adjacent to the alanine residues, which formed the inner core of the coiled-coils; alanines in the center were flanked by positively or negatively charged residues on either side.

These findings suggested that parallel coiled-coils will most like be formed and, hence, each of the negatively charged residues of one alpha-helix faces the positively charged residues of the neighboring alpha-helix, and *vice versa* (Figure 5B). On the basis of our analysis, one can speculate that the opposing charges play an important role in the formation of the coils [49], which supports the idea that the Alanine zipper constitutes a novel type of interaction domain.

### Homology model of the Alanine Zipper versus a Leucine Zipper

To address the possibility whether the Alanine zipper is able to mediate a stabile dimer conformation that is supported by the attraction from the opposing charges, we computed a three dimensional structural model of the AtBPC6 Alanine zipper. Therefore, we acquired the atomic coordinates from a monomeric crystal structure of the alpha-helical Leucine zipper of C-Jun (PDB ID: 1T2K) [55]. The positions of the conserved AtBPC6 Alanine zipper were homology modeled by superimposition and fitted onto the corresponding register of the conserved leucines in the C-Jun oncogene's zipper domain (Figure 5C and Supporting Figure S6). Besides the C-Jun and AtBPC6 zippers, we also modeled two mutant domain versions that provided us with more insight on the function of the alanines inside the zipper: In C-Jun-ALA the leucines that contribute to the zipper were exchanged for alanines, while in BPC6-LEU the conserved alanines inside the homology modeled AtBPC6 domain were replaced by leucines. Additionally, two domains were modeled in a shifted register of the helices (Supporting Figure S8): In BPC6-1 the conserved alanines of the native BPC6 domain sequence were fitted onto the ‘c’-position and in BPC6-3 onto the ‘a’-position of the C-Jun sequence.

The sidechains of the monomeric models were minimized for 200 steps and, subsequently, homodimeric models were obtained by structural alignments of the different monomers onto the crystal structure of the C-Jun-homodimer (PDB ID: 1JNM), followed by additional minimization steps.

A comparison of the homology model of the AtBPC6 Alanine zipper and the original C-Jun Leucine zipper (Figure 5D) already discloses differences between the opposing amino acid residues of the dimers and the distances within their helical backbones. In the C-Jun zipper domain the leucines are in close contact and form the hydrophobic core around which the two monomers are coiled and which is fully consistent with reports in literature [49], [50], [52], [55]. Inside the two facing AtBPC6 dimerization domain models, the conserved alanines are proximal to each other (Figures 5E & F), however, they appear not directly to be involved in the stabilization of the dimers and they do not form an obvious knobs-into-holes structure. Hence, the possible function of the conserved alanines of AtBPC6 dimerization domain might be the reduction of sterical hindrance due to the tiny methyl group side chain and, thus, to make way for the neighboring positively and negatively charged residues. Supportive of this assumption is the presence of proteins encoded in bacteria genomes with related domain structures, but with a glycine instead of alanines in the coiled-coil region (Supporting Figure S5), which indicates that only tiny amino acids are permissive at this position in the zipper.

As one consequence of small amino acids, the model of the group II BPC coiled-coils will likely have strands closer to each other and smaller in diameter when compared with the Leucine zipper homodimer structure of C-Jun (Supporting Figure S7).

Likewise, the diameter of the modeled C-Jun-ALA zipper mutant version appeared to be smaller, while it was larger for BPC6-LEU. Moreover, the leucines of the BPC6-LEU dimer form a hydrophobic core homologous to the knob-in-hole signature of the C-Jun Leucine zipper dimer [45], [49], [55].

These findings can be supported by RMSD of the backbone atoms of the dimeric homology models during a 19.5 ns production run, in which the structural stability of the models is compared with the actual C-Jun dimer structure.

As expected, the RMSD for AtBPC6 and C-Jun-ALA deviate more from the backbone coordinates than that of BPC6-LEU or the C-Jun during the first 10 ns (Supporting Figure S9). The register shifted version BPC6-3 was of comparable stability as the native AtBPC6 model. In contrast, the BPC6-1 model did not form any stabile conformation (Supporting Figure S9 and S10). The dimeric structure of BPC6-1 starts to disintegrate from the N-Terminus during equilibration. The distance between the C-alpha atoms of the N-TerminalGLU increases from 25.0 Angstroem(t0) to 34.6 Angstroem within 2.3ns of MD. After 2.3ns the distance between monomers varies but the monomers form nevers dimers again. The dimers are only connected via hydrogen bonds at the C-terminal moiety during the whole simulation. (Supporting Figure S10).

### Alanine Zippers form stable Dimers via Electrostatic Interaction

Besides the described observations, our approach allowed us to count the number of intermolecular salt bridges and hydrogen bonds that were formed between the two monomers and to assess, which of the residues were involved in those interactions.

The models disclosed that there are almost three times as many hydrogen bonds and almost six times as many salt bridges in the Alanine zipper dimer of AtBPC6 compared to the Leucine zipper dimers of C-Jun (Figures 5 G & H and Supporting Table S1). The register shifted BPC6-3 formed a stabile dimer with many hydrogen bonds and a comparable number of salt bridges as in the native BPC6 model (Supporting Table S1). This is interesting, as the known Alacoil zippers are characterized by conserved alanines in either ‘a’- or ‘d’-positions. As the BPC6 models in both of the registers form stable dimers, we can not make a definite decision on the register conformation.

Compared with AtBPC6 dimers, the *in silico* mutation of the conserved alanines to leucines in BPC6-LEU decreased the number of both hydrogen bonds and salt bridges (Supplementary Table S1).

In contrast, the replacement of leucine residues with alanines in the C-Jun-ALA monomers led to a loss of the known zipper structure and the polar hydrophobic residues that used to stabilize the hydrophobic core from the outside were turned to the inside of the coiled-coil (Supplementary Table S1) [49]. As a consequence, the known zipper structure was lost. This on the one hand increased the number of hydrogen bonds and salt bridges that were detected compared to the native C-Jun structure.

To gain further insight into the nature of the homodimeric interaction of the zippers, we used 5 ns of our 19.5 ns production run, where the structure of the protein backbone were relative stable, to approximate the binding free energies between our modeled structures (Supporting Figure S11). This should allow us to rank the binding affinities between the different coiled-coil structures by their binding free energies.

On the basis of its homodimeric crystal structure the computed ΔG_Binding_ for the C-Jun homodimers was −22.24 kcal mol^−1^ (Supplementary Table S2).

The binding free energy for the homology modeled AtBPC6 dimer was nearly twice as big compared with C-Jun (ΔG_Binding_ = −44.62 kcal mol^−1^) and, hence, can explain the stable dimer formation of the two Alanine zipper monomers *in vivo*. Comparable values were found for the register shifted BPC6-3 dimer (ΔG_Binding_ = −39.82 kcal mol^−1^) and, therefore, no definite conclusion on the ‘a’ or ‘d’ register of the Alanines can be drawn.

For the mutated BPC6-LEU dimer a ΔG_Binding_ of −46.85 kcal mol^−1^ was calculated; a value equivalent to that of the wildtype AtBPC6 Alanine zipper. This finding suggests that a chimera of an Alanine and Leucine zipper by introducing a hydrophobic core into the Alanine zipper's electrostatic backbone structure would not necessarily increase the strength of binding (Supplementary Table S2).

The C-Jun-ALA zipper was more unstable and the computed ΔG_Binding_ was 5.02 kcal mol^−1^. This had been expected, as the hydrophobic core between the monomers was removed with the mutation of the leucines.

### Group II BBR/BPC proteins form parallel Dimers *in planta*

**Figure 6:**
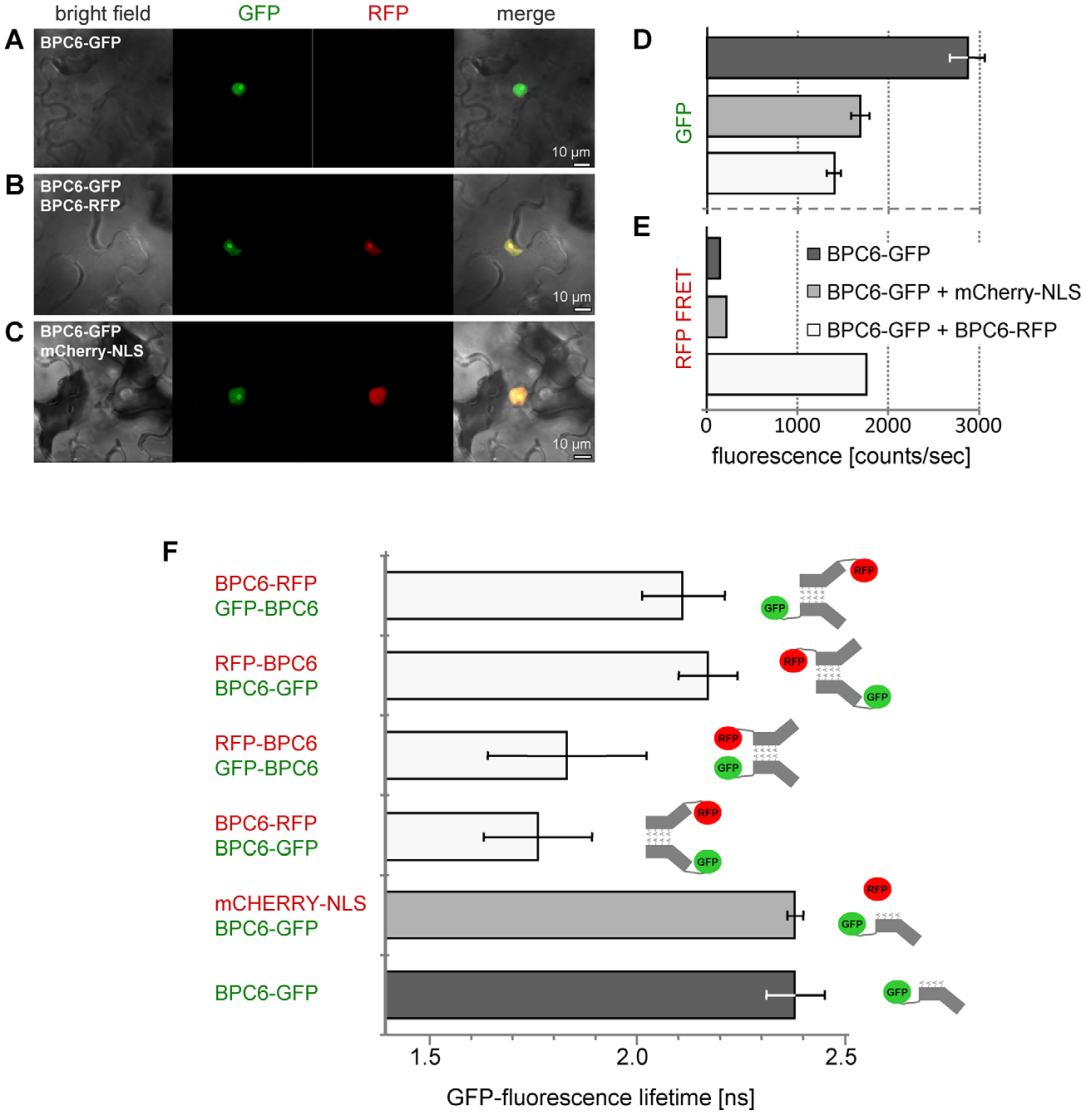
AtBPC6 forms parallel dimers *in planta*. (**A–C**) Confocal laser scanning microscopy analysis of GFP-/RFP-fusion proteins and mCherry-NLS in transiently co-transformed *Nicotiana benthamiana* epidermis cells. All proteins localize to the nucleus. GFP fluorescence intensities (**D**) and RFP-FRET fluorescence intensities (**E**) under GFP-excitation light. (**F**) *In vivo* GFP fluorescence life time measurements of all four possible GFP/RFP protein combinations fused to either the N- or the C-terminus of AtBPC6. BPC6-GFP and BPC6-GFP/mCherry-NLS combinations serve as controls. Pictographs (right hand side) display the only possible zipper orientations that are in accordance with the GFP fluorescence life time measurements.

On the basis of our initial assumption that BBR/BPC type Alanine zippers share similarities to Leucine zippers, we postulated parallel dimer formation of AtBPC6 (Figure 5 I), which was supported by our homology models. To validate our previous findings, we applied the powerful technique of fluorescence resonance energy transfer (FRET) combined with two-chromophore fluorescence lifetime imaging microscopy (FLIM) [56], [57]. Our custom made FLIM system consisted of a confocal sample scanning microscope (CSSM), a spectral integrating detector for measuring fluorescence intensities and a time-correlated single-photon counting board for recording fluorescence lifetime decay [58], [59]. GFP- and RFP-AtBPC6 fusion proteins serve as excitation energy donor (GFP) or acceptor (RFP) and were transiently co-expressed in *Nicotiana benthamiana* epidermis cells. The fluorescent proteins were fused to either the N- or the C-terminus of AtBPC6 and analyzed for GFP- or RFP-emission under GFP-excitation light. GFP-fluorescence intensities of cells transformed with GFP-BPC6 alone or co-transformed with mCherry-NLS served as references. The presence of the fusion proteins in the nucleus was verified by conventional confocal laser scanning microscopy (Figure 6 A–C).

While strong GFP-fluorescence was recorded for all transformed cells (Figure 6 D), under GFP-excitation light RFP-FRET-fluorescence could only be measured for BPC6-GFP/BPC6-RFP (Figure 6 E) or GFP-BPC6/RFP-BPC6 co-transformed cells (data not shown). These findings are already a strong indication that parallel AtBPC6 dimers are formed *in planta*.

An antiparallel orientation of the zipper would arrange the chromoproteins for BPC6-GFP/BPC6-RFP (both fusions at the C-terminus) further apart than those for GFP-BPC6/RFP-BPC6 (both fusions at the N-erminus). We, therefore, analyzed the lifetime of GFP-fluorescence, which provides information about the physical and chemical environment of the chromoprotein. The closer RFP (FRET-acceptor) localized to GFP, the more decrease of the GFP-lifetime will be seen. The measurements revealed significant differences (*p*<0.01) for all co-transformed cells compared to the control transformations (Figure 6 F). The combinations BPC6-GFP/BPC6-RFP and GFP-BPC6/RFP-BPC6 displayed strongest decrease in GFP-lifetime. The values for the two co-transformations did not differ significantly between the two samples. Hence, this data can only be explained by a parallel orientation of the helices.

Consistently, the combinations BPC6-GFP/RFP-BPC6 and GFP-BPC6/BPC6-RFP exhibited a much lower affect on the GFP-lifetime (Figure 6 F). We expected the strongest GFP-lifetime decrease for these later combinations for antiparallel topology of the AtBPC6 Alanine zipper.

Taken together, the *in planta* data validates our homology models. These findings have a crucial implication for AtBPC6 function: The helices are oriented in parallel towards each other, which proposes a model in which the DNA-binding domains of the dimers make contact to neighboring GAGA-motifs on the same side of the DNA-helix. This is in accordance with earlier analyses on the distribution of BBR/BPC binding sites in the core-promoter sequences, where GA- and TC-rich repeats are found in close proximity to each other at the transcriptional start sites of the genes [60], [61].

### Concluding remarks

Since the initial identification and characterization of GAGA-binding proteins in plants, only little progress has been made towards a functional elucidation of these proteins. It was shown that the BBR/BPC proteins are capable of DNA-bending *in vitro* and it has been assumed they might harbor an orthologous function to Drosophila Trl or Psq *in planta* [13], [18].

A prerequisite for GAGA-binding factor action in animals is the formation of multimers and higher order complexes, which affect DNA-condensation. In this work, we demonstrate that BBR/BPC proteins are forming stabile dimers in the plant nucleus and nucleolus, which is required for a possible functional orthology to Trl and Psq.

Moreover, we demonstrate that group II BBR/BPC dimerization is encompassed by a novel Alanine zipper protein-protein interaction domain, which mediates binding of parallel oriented helical bundles *via* electrostatic interaction.

We suggest a model in which the two major groups of BBR/BPC proteins act in a supposedly competitive manner through binding to the same DNA-motifs inside the transcriptional units of genes, but fulfilling distinct functions. Most likely, the BBR/BPC proteins form dimers or multimers to influence the transcriptional activation by DNA-bending, similar to what has been reported for their *Drosophila* counterparts Trl or Psq.

## Materials and Methods

### Cloning and sequence analysis of AtBPC6 orthologs

Arabidopsis thaliana Columbia-0 sequence of AtBPC6 [NCBI: ABL67949] was used for all analyses. Genomic DNA of *Olimarabidopsis pumila* and *Cardamine pratensis* was extracted using a modified Edwards buffer [62]. Gradient TAIL-PCR was performed using Arabidopsis specific primers (BPC6-ATG-S1: ATGGATGATGGTGGGCATCG; BPC6-woSTOP-A1: TTTAATCGTAATGTAGCGG) in combinations with random nonamers. Amplicons were cloned into pCR-Topo (Invitrogen) and sent for sequencing at 4baseLab (Reutlingen). Searches with Blast [63] against the EST database identified the introns and coding exons. Sequences have been submitted to GenBank [NCBI: ABC25623 and NCBI: ABG57062].

Conceptual translation of the ORFs into amino acid sequence was done to compute a multiple alignment with ClustalW [64].

### Transient transformation of tobacco leaf cells

*Nicotiana benthamiana* plants were cultivated in the greenhouse (temperature: day 25°C/night 19°C, humidity 60%, photoperiod: 14 h).

*Agrobacterium tumefaciens* GV3101 pMP90 was infiltrated into the adaxial side of leaves from 5 week old tobacco plants as has been described [65]. Co-expression of p19 protein from tomato bushy stunt virus was used for suppression of transgene silencing [66], [67]. Transformation of several expression vectors was performed as described [68]. Transformed leaf areas were analyzed one to two days post infiltration.

### GFP/RFP fluorescence analysis and flow cytometry

All clones were constructed in Gateway™ (Invitrogen) compatible vectors. Clones containing the ORF of full-length or partial AtBPC6 sequences were established in pENTR-D-Topo (Invitrogen). For localization analysis of fusion proteins, the coding sequences were subsequently LR recombined to appropriate pUGT-DEST vectors. After recombination of the cDNA, the binary pUGT-DEST vectors express fusion proteins with either GFP or RFP at their N- or their C-terminus under the control of the Arabidopsis *Ubiquitin10*-promoter.

GFP fluorescence was analyzed in transiently transformed *Nicotiana benthamiana* epidermis cells using Confocal Laser Scanning Microscopy (CLSM).

For quantitative fluorescence analysis of plant nuclei by flow cytometry, leaf samples were incubated in glass Petri dishes and covered with extraction buffer (15 mM Tris, pH 7,5; 2 mM EDTA; 0,5 mM Spermine; 80 mM KCl; 20 mM NaCl; 15 mM ß-Mercaptoethanol; 0,1% Triton). Tissue was cut in small pieces and filtered through 40 µm sieve. Propidium iodide to a final concentration of 50 µg/ml was added to the nuclei suspension and incubated 5 min prior the analysis.

Flow cytometric analysis was performed with a MoFlo (Beckman-Coulter). PI and GFP were excited with an argon laser 488 nm (50 mW) and fluorescing nuclei were identified as the PI bound fraction in the PI Fluorescence (613/618) *vs.* GFP Fluorescence (530/540) plot.

### Bimolecular fluorescence complementation (BiFC) assay

BiFC was performed in transiently transformed *Nicotiana benthamiana* leaf epidermis cells [65]. For *in vivo* interaction analysis, the coding sequence inserts in pENTR-D-Topo (Invitrogen) vectors were LR recombined to either pSpyNe-GW or pSpyCe-GW binary destination vectors, which express fusion proteins with YFP-fragments at their N-termini [65].

Presence of YFP fluorescence was scored one to two days post infiltration using Confocal Laser Scanning Microscopy (CLSM).

### Yeast-two-hybrid analysis

Matchmaker™ (Clontech) compatible pGBKT7-DEST and pGADT7-DEST vectors were LR recombined with corresponding Entry clones and transformed to PJ69-4A yeast strain [66]. Complementation of auxotrophy was score by growth on selective media (CSM-L-,W-, Ade-). Enzymatic activity of the β-galactosidase reporter has been quantified by triple measurements of MUG substrate assays of three independent transformations [n = 9] and normalized to total protein amount [69]. Statistical background for no significant interaction has been calculated from enzymatic activity of empty AD-clone combinations.

### Optical and spectroscopic measurements

The FRET-FLIM measurements were performed with a custom-built CSSM (confocal stage scanning microscope), based on a Zeiss Axiovert 135 TV, and equipped with a pulsed supercontinuum laser–source (***SuperK***™, NKT Photonics) as excitation light source operating at 471 nm and a repetition rate of 40 MHz. A microscope objective with high numerical aperture (Plan-Neofluar, 100×/1.30 oil, Zeiss) was used to focus the excitation light as well as to collect the fluorescence emission. The setup was equipped with a 500 nm dichroic mirror (FF500-Di01-25×36, Semrock) to block back-scattered excitation light and with a 527 nm bandpass filter (Semrock BrightLine BL527/20) to detect GFP-fluorescence. An avalanche photo diode (PDM series, MicroPhotonDevices (MPD), Italy) served as a spectrally integrating detector to record fluorescence intensity. Lifetime decays were recorded using a time-correlated single photon counting board (PicoHarp 300, Picoquant, Software: SymPhoTime, Picoquant) for data acquisition and the MPD as a detector. The intensity decay curves were fitted by a monoexponential decay function, which was convolved from the instrument response function (IRF) measured without the long pass filter to record back-reflected excitation light.

### Structural modeling

On the basis of the crystal structures of the coiled-coil dimerization domain of the bZIP transcription factor C-Jun (PDBID: 1T2K and 1JNM) structural models of the dimerization domain of BPC6 were created for the monomers and dimers. Using PyMOL (http://www.pymol.org), the protein sequence from residue 37 to 81 was fitted onto the structure of the C-Jun dimerization domain (residue 278 to 322). Thereby the alanine residues of BPC6 were superposed onto the corresponding leucine residues at the ‘d’ positions of the alpha-helical zipper domain of C-Jun. The models of the register shifted BPC6-1 and BPC6-3 were constructed using PyMOL. The protein sequence of BPC6 was modeled onto the residues 268–308 (BPC6-1) and 266–306 (BPC6-3) of the c-Jun crystal structure. The dimeric models were minimized for 2000 steps to remove structural clashes using the conjugate gradient algorithm. Force field parameters for the protein were taken from the AMBER all-atom protein force field ff03 [70].

### Molecular Dynamics (MD) simulation

The molecular dynamics simulations were performed with the SANDER and PMEMD module of AMBER 9. Every dimeric structure of BPC6, C-Jun, BPC6-LEU, C-Jun-ALA, BPC6-1 and BPC6-3 was placed in a rectangular water box composed of TIP3P water molecules [71] with a buffering distance of 12 Å around the protein, using Leap (AmberTools 1.4). Periodic boundary conditions in all dimensions were used for the simulation of the systems. The dimensions of the periodic boxes (X;Y;Z) were 55.366Å ;51.067 Å ;97.698 Å for BPC6, 55.366 Å; 51.067 Å; 98.258 Å for BPC6-LEU, 49.439 Å; 50.192 Å; 90.441 Å for BPC6-1, 54.288 Å;47.888 Å; 92.658 Å for BPC6-3, 56.742 Å; 55.386 Å; 99.174 Å for C-Jun and C-Jun-ALA. Counterions were added to maintain the electroneutrality. The four different systems consist out of 21'439 atoms (BPC6), 24'521 atoms (C-Jun), 21'631 atoms (BPC6-LEU), 24'461 atoms (C-Jun-ALA), 20'515 atoms (BPC6-1) and 21'901 atoms (BPC6-3). Long-range electrostatic interactions were estimated using the particle mesh Ewald method [72]. Bonds involving protons were constrained with the SHAKE algorithm [73] using the default tolerance (1.0×10^−5^Å) and time steps of 2fs. Non-bonded interactions were truncated at 12Å and the non-bonded list was updated every 25 steps. Each system was minimized for 500 steps using the steepest descent algorithm followed by 500 steps with the conjugate gradient algorithm. The systems were gradually heated from 0.1 to 300K by 50-ps constant volume dynamics (NVT ensemble). The Langevin dynamics with a collision frequency of 2.0 ps^−1^ was applied to control the temperature of the system. The density of the system was adjusted to 1 g/cm^3^ by 50ps by constant pressure (1atm) and temperature dynamics (NPT ensemble). A pressure relaxation time of 2.0ps was used. During the minimization, heating and equilibration all atoms of the complex were restrained to their initial positions by a weak force constant of 2 kcal mol^−1^ * Å^−2^. A subsequent unconstraint 19.5ns production run was performed at constant pressure (1atm) and constant temperature (300K). Coordinates for analysis were saved every 1ps. The trajectory was analyzed with the ptraj module from AMBER. Root-mean-square deviations (RMSD) of the backbone atoms from the different protein structures were computed from the MD trajectory relative to the initial structures to estimate the stabilization of the systems (Supporting Figures S8 and S9).

### Estimation of binding energies

Binding free energies between the dimers were estimated using the single molecular dynamics trajectory method and the MM-PBSA [74] and normal mode analysis. For the calculation of the enthalpic contribution, multiple snapshots of the ligand, receptor and the ligand-receptor complex were extracted every 10 ps from a 5ns period of the MD trajectories were the backbone RMSD was relatively stable (Supporting Figure S9). Since estimation of entropic contribution is more time consuming, snapshots for this calculation were extracted every 100ps from the same time windows. All water and counter ions were stripped. To calculate the binding free energies of 500 snapshots, which were averaged afterwards, the following formula was used:
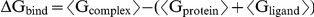
The free energy terms include the contributions from gas phase, solvation and entropic effects and were calculated by using the formula below:
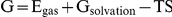


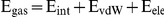


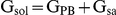
where E_gas_ is the sum of the internal energies (bond, angle and torsion) and the van der Waals and electrostatic energies.

The polar component of the solvation free energy (G_PB_) was calculated by using the PBSA program in AMBER9.0. The dielectric constant for the solute (inside the solute) was set to 1 and 80 in the solvent in this work. The non-polar component (G_sa_) was determined with
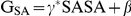
in which SASA (solvent accessible surface area) was calculated with *molsurf*. The values for γ and β were set to 0.0072 kcal mol^−1^ Å^−2^ and 0 kcal mol^−1^ , respectively. The contribution of entropy (TS) to binding free energies, which arises from changes of the translational, rotational and vibrational degrees of freedom, was calculated by normal mode analysis with the nmode module of AMBER 9.

### Image capture and analysis

CLSM was performed using a Leica TCS SP2 confocal microscope (Leica Microsystems GMBH). CLSM image capture and analysis was described previously [66].

### Sequence analysis

Sequences acquired from GenBank accessions: AtBPC1 [NCBI: AAM15473], AtBPC4 [NCBI: AAM15408], AtBPC6 [NCBI: ABL67949], rice Os06g0130600 [NCBI: AAX59046], Physcomitrella [NCBI: BJ586982], Solanum [NCBI: BT013886] Medicago [NCBI: ABL10372].

Multiple sequence alignments were computed with ClustalW [75].

Secondary structure prediction was performed with SOPMA (http://npsa-pbil.ibcp.fr/cgi-bin/npsa_automat.pl?page=npsa_sopma.html) on the basis of multiple sequence alignments [76].

Analysis of the coiled-coil domain was performed with the following programs: COILS (http://www.ch.embnet.org/software/COILS_form.html) [52], Paircoil2 [53] and MultiCoil [77].

## Supporting Information

Figure S1 **Secondary structure prediction of AtBPC6.**
Prediction of AtBPC6 protein secondary structure using SOPMA (http://npsa-pbil.ibcp.fr/cgi-bin/npsa_automat.pl?page=npsa_sopma.html). For orientation, schematic positions of the coiled-coil domain (checked-grey), nuclear localization signal (dark grey) and zinc-finger like DNA-binding domain (black) are shown. Raw probability scores for helix, sheet, turn or coil secondary structures in a sliding window of 17 amino acids.
(TIF)

Figure S2 **Relative signal intensities of DAPI- and GFP-fluorescence in the nucleus and nucleolus.**
Relative signal intensities of DAPI- and GFP-fluorescence in nuclei are measured from laser confocal microscopy images of BPC6-GFP, BPC6-NLS-GFP and mGFP expressed in *Nicotiana benthamiana* epidermis cells (Figure 1D). Fluorescence intensities (y-axis) of BPC6-GFP and BPC6-NLS-GFP overlap with DAPI in the nucleoplasm, but are highest at the periphery of the nucleoli.
(TIF)

Figure S3 **Subcellular localization of GFP-fusion proteins of AtBPC6 fragments.**
Laser confocal microscopy analysis of GFP fusion proteins and free mGFP in transiently transformed *Nicotiana benthamiana* epidermis cells. All hybrid fusion proteins of AtBPC6 fragments containing the entire 31 amino-acid long NLS localize to the nucleus and the nucleolus.
(TIF)

Figure S4 **Prediction of the coiled-coil region present in group II BPC proteins.**
Prediction of a coiled coil containing domain at the N-terminus of AtBPC6 has been performed with 5 programs independently (Coils; Paircoil; Paircoil2; Multicoil; 2ZIP). All programs predict an extended α-helical coiled-coil region, which does not resemble topological features characteristic for Leucine zipper-like coils. The output of the program Coils (http://www.ch.embnet.org/software/COILS_form.html) is displayed, accompanied by a schematic overview of AtBPC6 domains and the primary sequence forming the coiled coil, respectively. alanines with an evenly spacing of 7 amino acids are highlighted in red.
(TIF)

Figure S5 **Presence of BPC-like coiled-coil regions outside the BPC-family.**
BPC-like coiled-coil regions from green plants, brown algae, cyanobacteria, bacteria, fungi and animals are aligned. alanines with an evenly spacing of 7 amino acids are highlighted by black background color. Not perfectly matching alanines that still contribute to a possible zipper structure are given in bold face.
All sequences displayed are predicted to form coiled-coils (*p*≥0.8) without Leucine zipper-like topology. Sequences were retrieved from GenBank: *Ostreococcus lucimarinus* CCE9901 predicted protein (OSTLU_18871) [GenBank: XM_001422391], *Desmarestia viridis* cytochrome oxidase subunit II [GenBank: AAS79051], *Physcomitrella patens* CHUP1A mRNA for chloroplast unusual positioning 1A [GenBank: AB292414], *Microcystis aeruginosa* NIES-843 [GenBank: AP009552], *Anabaena variabilis* [GenBank: ABA21837], *Burkholderia pseudomallei* strain K96243 [GenBank: BX571965], *Azorhizobium caulinodans* [GenBank: ABA21837], *Coprinopsis cinerea* hypothetical protein (CC1G_08107) [GenBank: XM_001836670], *Magnaporthe oryzae* hypothetical protein (MGG_04186) [GenBank: XM_361712], *Phaeosphaeria nodorum* [GenBank: XM_001801867], *Saccharomyces cerevisiae* RPL7B [GenBank: NM_001184012], *Aspergillus fumigatus* NACHT domain protein [GenBank: XM_750964], *Homo sapiens* DLG5 [GenBank: BC146794], *Sus scrofa* similar to microtubule-associated protein 1A [GenBank: XM_001925969], *Mus musculus* SMC1A [GenBank: AK017948], *Homo sapiens* SMC1A [GenBank: BC080185]; Sequences with aberrant Alanine zipper signatures are *Homo sapiens* LUZP4 [GenBank: BC080185], *Burkholderia cenocepacia* hypothetical protein [GenBank: YP_002232335]. Grey background indicates variation in positioning of the positively or negatively charged residues within the aberrant Alanine zipper signatures.
(TIF)

Figure S6 **Homology model of alternating amino acids in the AtBPC6 Alanine zipper.**
The homology model of the coiled-coil structure of AtBPC6 was computed by using the backbone coordinates of the C-Jun Leucine zipper. For better visualization of the alternating amino acid residues inside the Alanine zipper region were color coded: blue -positive charged; red - negative charged; yellow – conserved alanines; green – all other amino acids. (A) and (B) illustrate the identical model from angles as indicated. The molecules were fitted and displayed by using PyMOL (http://www.pymol.org).
(TIF)

Figure S7 **Homology models of monomeric and homodimeric coiled-coil structures.**
The homology models of the monomeric and homodimeric coiled-coil regions of BPC6, C-Jun, C-Jun-Ala and BPC6-Leu were computed by using the backbone coordinates of the C-Jun Leucine-zipper. Conserved alanine and leucine residues (both at ’d’ position of the register) in BPC6 and C-Jun or mutated alanines and leucins in BPC6-Leu or C-Jun-Ala were highlighted in yellow. The figure illustrates the identical models from two angles as monomers or homodimers. The monomeric molecules were fitted by using PyMOL (http://www.pymol.org), the dimers were subsequently computed and displayed using AMBER (http://ambermd.org/).
(TIF)

Figure S8 **Alignment of the native AtBPC6 and the register shifted BPC versions with C-Jun.**
The register of the C-Jun alpha-helix is shown on top of each alignment. Conserved alanine or leucine residues are highlighted in red and yellow background. The native sequences are aligned to fit the conserved amino acids at ‘d’-position of the register. In BPC6-1 the register is shifted by one position, in BPC6-3 it is shifted by three positions, respectively. Thus, the conserved alanines are now at positions ‘c’ (BPC6-1) or ‘a’ (BPC6-3).
(TIF)

Figure S9 **RMSD of the backbone atoms of the dimeric models during the production run (19.5ns).**
The 19.5ns production run was performed at constant pressure and constant temperature. Coordinates for analysis were saved every 1 ps. Root-mean-square deviations (RMSD) of the backbone atoms of the six indicated protein structures were computed from the MD trajectory relative to the initial structures.
(TIF)

Figure S10 **Homology model of the register shifted BPC6-1.**
The homology model of the coiled-coil structure of AtBPC6 was computed by using the backbone coordinates of the C-Jun Leucine-zipper, but shifted by −1 register. The conserved alanine residues in BPC6 at ‘d’-position are not at ‘c’-position. Note that within the short period of 2.3ns the two helices departed. (**A**) start position; (**B**) 2.3ns of equilibration.
(TIF)

Figure S11 **RMSD of the backbone atoms of the dimeric models during the time period that was used for the calculation of the binding free energies.**
Schematic overview of the 5 nanosecond periods from the RMSD of the backbone atoms that were taken for the calculation of ΔG_Binding_: BPC6 - 15.5ns to 19.5ns (black line); C-Jun - 11.0ns to 16.0ns (red line); BPC6-LEU - 15.5ns to 19.5ns (green line); c-Jun-ALA - 11.0ns tp 16.0ns (violett line); BPC6-3 - 15.5ns to 19.5ns (cyan).
(TIF)

Table S1 **Hydrogen bonds and salt bridges that were formed between the two monomers during the 19.5ns production run.**
The table gives the pairs of amino acids and their positions within the zipper domains. Only those amino acids of one monomer are listed that have formed either hydrogen bonds or salt bridges with the respective pairing residue of the other monomer.
(XLS)

Table S2 **Binding free energy components of the dimmers.**
The calculated binding free energies are listed. Values have been inferred from 5 ns of 19.5ns production run and are given in kcal mol^−1^.
(XLS)
