# Supplementary figures and images for: Alanine Zipper-Like Coiled-Coil Domains Are Necessary for Homotypic Dimerization of Plant GAGA-Factors in the Nucleus and Nucleolus
Source: PLoS One. 2011 Feb 10;6(2):e16070. doi: 10.1371/journal.pone.0016070 (PMC3037368; doi:10.1371/journal.pone.0016070)

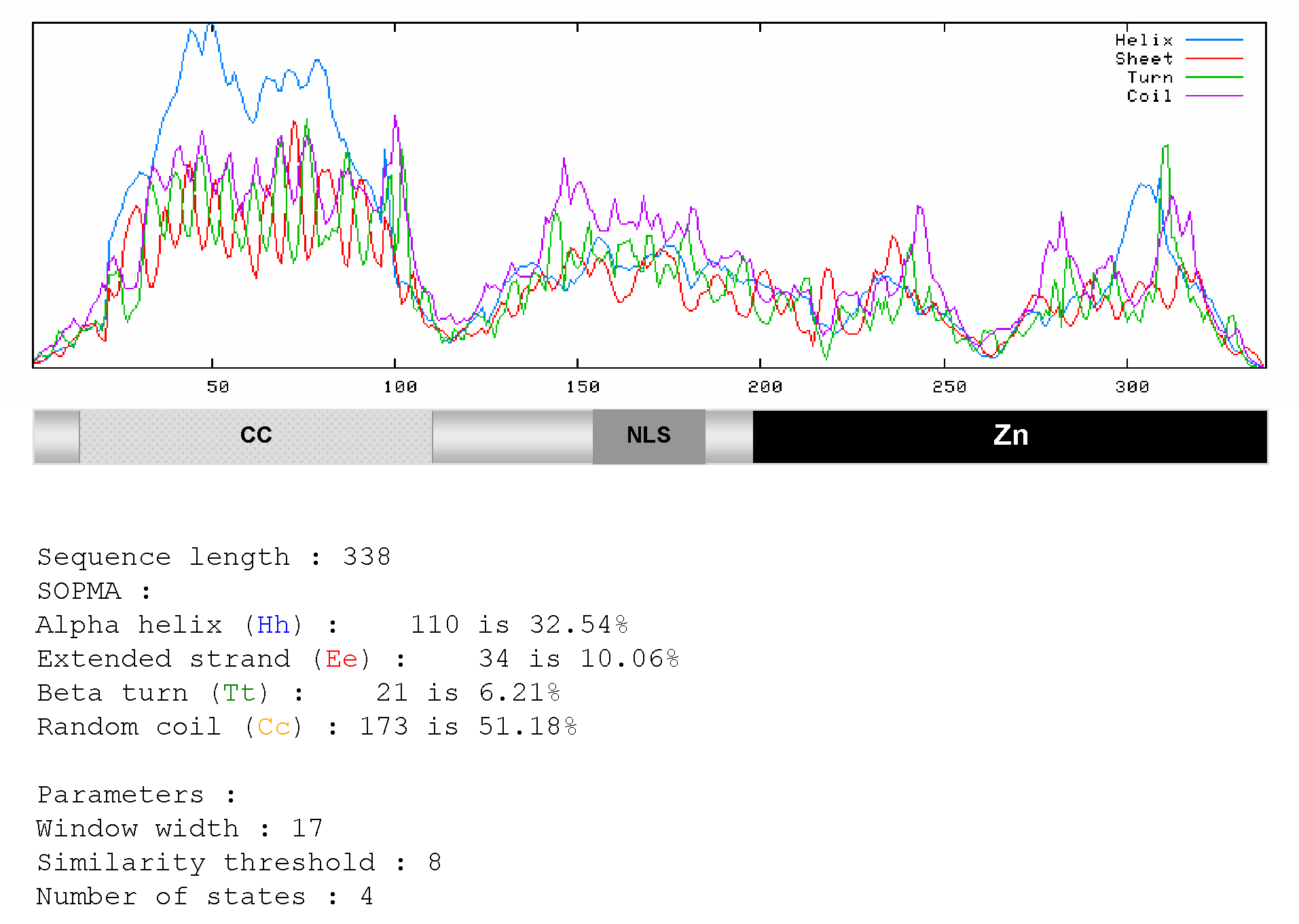

Supplement: Figure S1 — Secondary structure prediction of AtBPC6. Prediction of AtBPC6 protein secondary structure using SOPMA (http://npsa-pbil.ibcp.fr/cgi-bin/npsa_automat.pl?page=npsa_sopma.html). For orientation, schematic positions of the coiled-coil domain (checked-grey), nuclear localization signal (dark grey) and zinc-finger like DNA-binding domain (black) are shown. Raw probability scores for helix, sheet, turn or coil secondary structures in a sliding window of 17 amino acids. (TIF) [file pone.0016070.s001.tif]

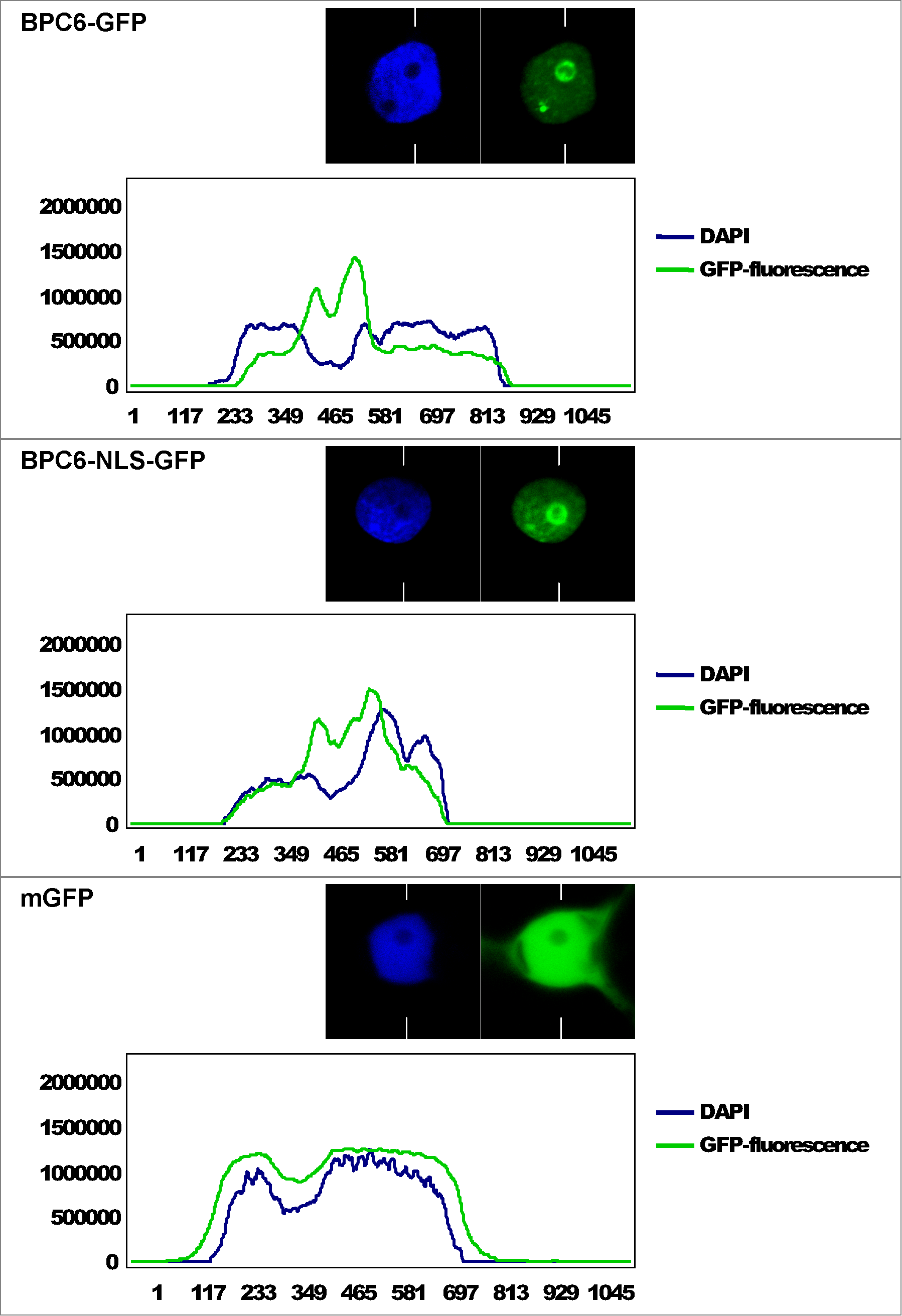

Supplement: Figure S2 — Relative signal intensities of DAPI- and GFP-fluorescence in the nucleus and nucleolus. Relative signal intensities of DAPI- and GFP-fluorescence in nuclei are measured from laser confocal microscopy images of BPC6-GFP, BPC6-NLS-GFP and mGFP expressed in Nicotiana benthamiana epidermis cells (Figure 1D). Fluorescence intensities (y-axis) of BPC6-GFP and BPC6-NLS-GFP overlap with DAPI in the nucleoplasm, but are highest at the periphery of the nucleoli. (TIF) [file pone.0016070.s002.tif]

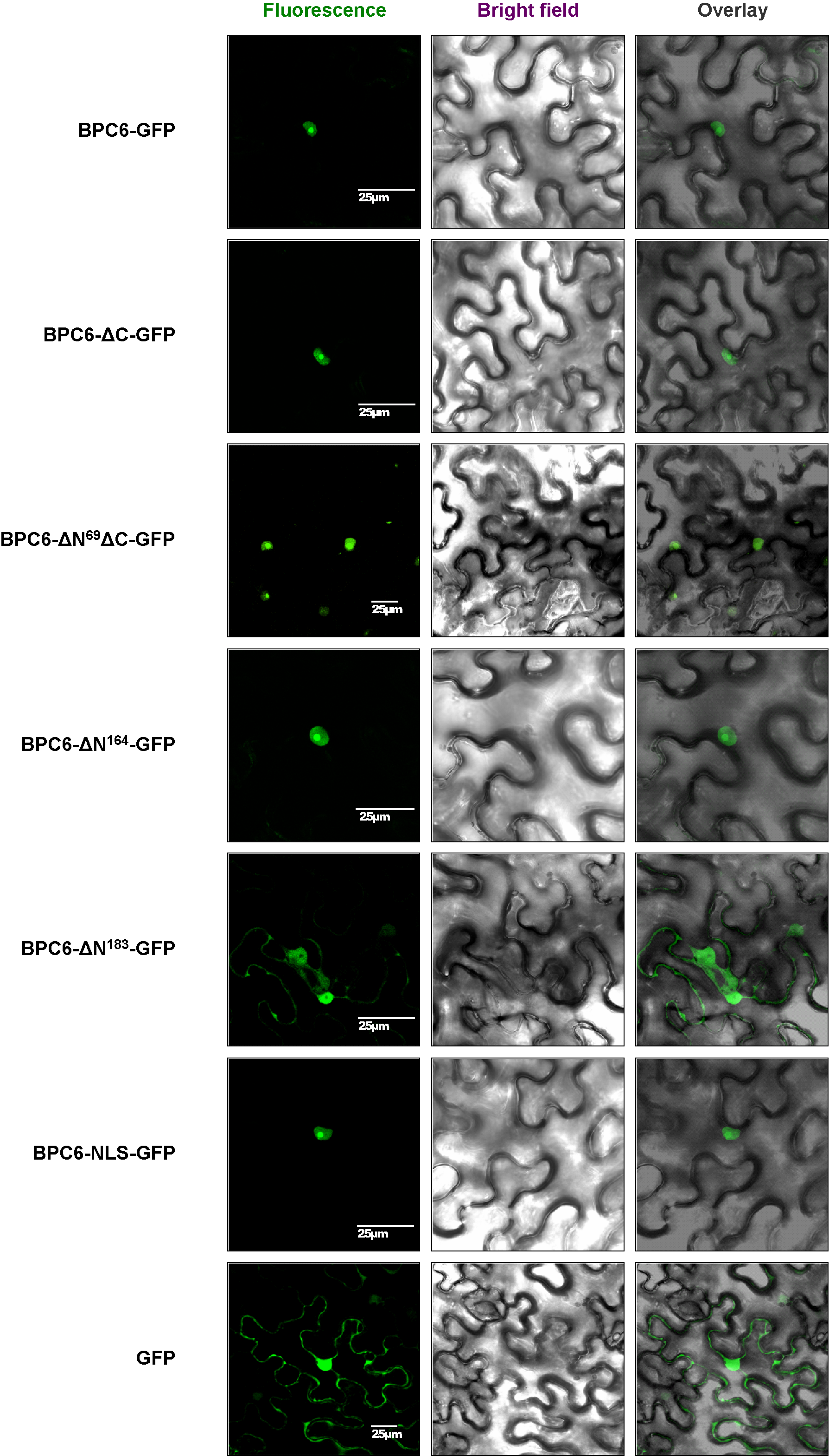

Supplement: Figure S3 — Subcellular localization of GFP-fusion proteins of AtBPC6 fragments. Laser confocal microscopy analysis of GFP fusion proteins and free mGFP in transiently transformed Nicotiana benthamiana epidermis cells. All hybrid fusion proteins of AtBPC6 fragments containing the entire 31 amino-acid long NLS localize to the nucleus and the nucleolus. (TIF) [file pone.0016070.s003.tif]

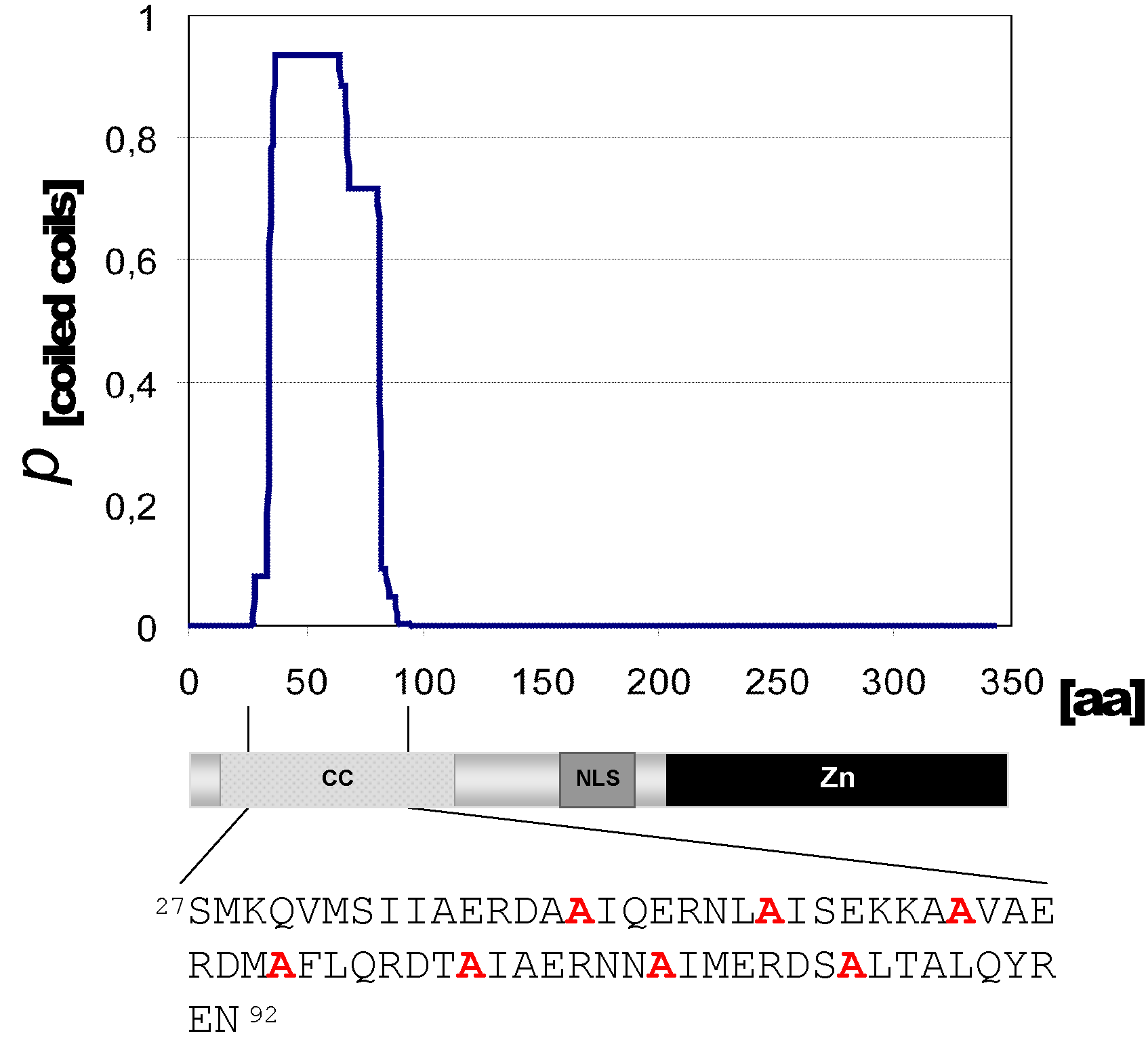

Supplement: Figure S4 — Prediction of the coiled-coil region present in group II BPC proteins. Prediction of a coiled coil containing domain at the N-terminus of AtBPC6 has been performed with 5 programs independently (Coils; Paircoil; Paircoil2; Multicoil; 2ZIP). All programs predict an extended α-helical coiled-coil region, which does not resemble topological features characteristic for Leucine zipper-like coils. The output of the program Coils (http://www.ch.embnet.org/software/COILS_form.html) is displayed, accompanied by a schematic overview of AtBPC6 domains and the primary sequence forming the coiled coil, respectively. alanines with an evenly spacing of 7 amino acids are highlighted in red. (TIF) [file pone.0016070.s004.tif]

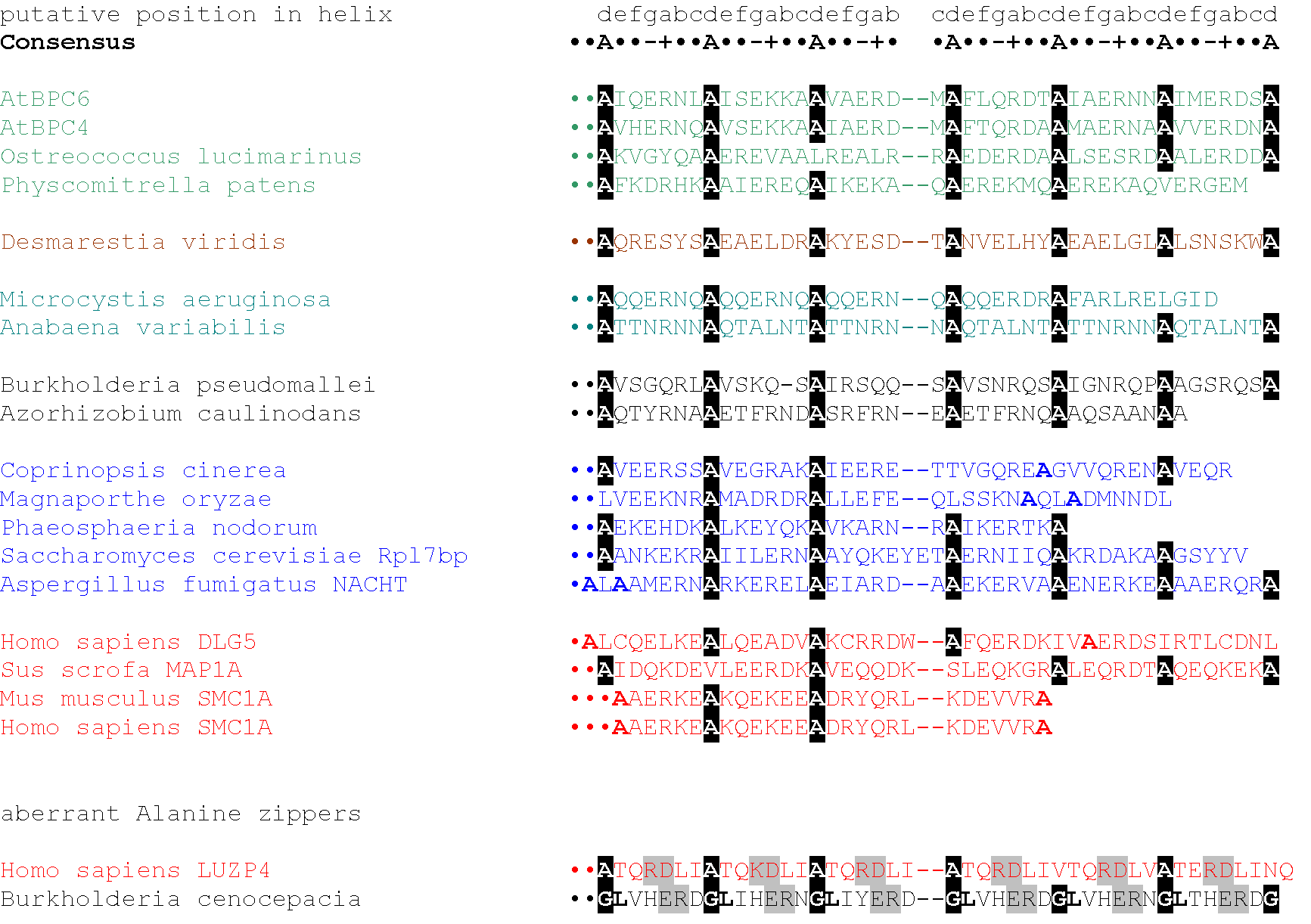

Supplement: Figure S5 — Presence of BPC-like coiled-coil regions outside the BPC-family. BPC-like coiled-coil regions from green plants, brown algae, cyanobacteria, bacteria, fungi and animals are aligned. alanines with an evenly spacing of 7 amino acids are highlighted by black background color. Not perfectly matching alanines that still contribute to a possible zipper structure are given in bold face. All sequences displayed are predicted to form coiled-coils (p≥0.8) without Leucine zipper-like topology. Sequences were retrieved from GenBank: Ostreococcus lucimarinus CCE9901 predicted protein (OSTLU_18871) [GenBank: XM_001422391], Desmarestia viridis cytochrome oxidase subunit II [GenBank: AAS79051], Physcomitrella patens CHUP1A mRNA for chloroplast unusual positioning 1A [GenBank: AB292414], Microcystis aeruginosa NIES-843 [GenBank: AP009552], Anabaena variabilis [GenBank: ABA21837], Burkholderia pseudomallei strain K96243 [GenBank: BX571965], Azorhizobium caulinodans [GenBank: ABA21837], Coprinopsis cinerea hypothetical protein (CC1G_08107) [GenBank: XM_001836670], Magnaporthe oryzae hypothetical protein (MGG_04186) [GenBank: XM_361712], Phaeosphaeria nodorum [GenBank: XM_001801867], Saccharomyces cerevisiae RPL7B [GenBank: NM_001184012], Aspergillus fumigatus NACHT domain protein [GenBank: XM_750964], Homo sapiens DLG5 [GenBank: BC146794], Sus scrofa similar to microtubule-associated protein 1A [GenBank: XM_001925969], Mus musculus SMC1A [GenBank: AK017948], Homo sapiens SMC1A [GenBank: BC080185]; Sequences with aberrant Alanine zipper signatures are Homo sapiens LUZP4 [GenBank: BC080185], Burkholderia cenocepacia hypothetical protein [GenBank: YP_002232335]. Grey background indicates variation in positioning of the positively or negatively charged residues within the aberrant Alanine zipper signatures. (TIF) [file pone.0016070.s005.tif]

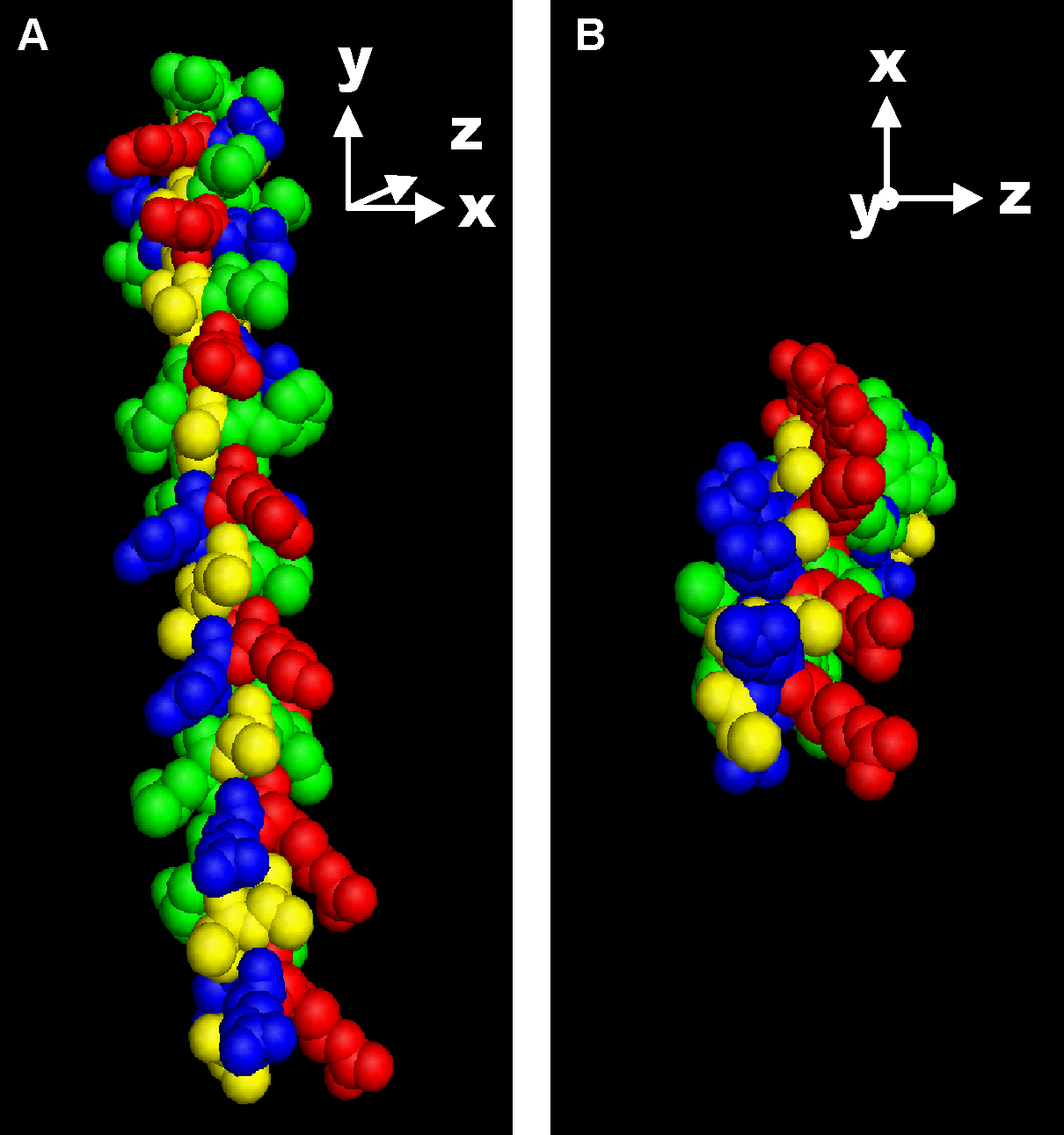

Supplement: Figure S6 — Homology model of alternating amino acids in the AtBPC6 Alanine zipper. The homology model of the coiled-coil structure of AtBPC6 was computed by using the backbone coordinates of the C-Jun Leucine zipper. For better visualization of the alternating amino acid residues inside the Alanine zipper region were color coded: blue -positive charged; red - negative charged; yellow – conserved alanines; green – all other amino acids. (A) and (B) illustrate the identical model from angles as indicated. The molecules were fitted and displayed by using PyMOL (http://www.pymol.org). (TIF) [file pone.0016070.s006.tif]

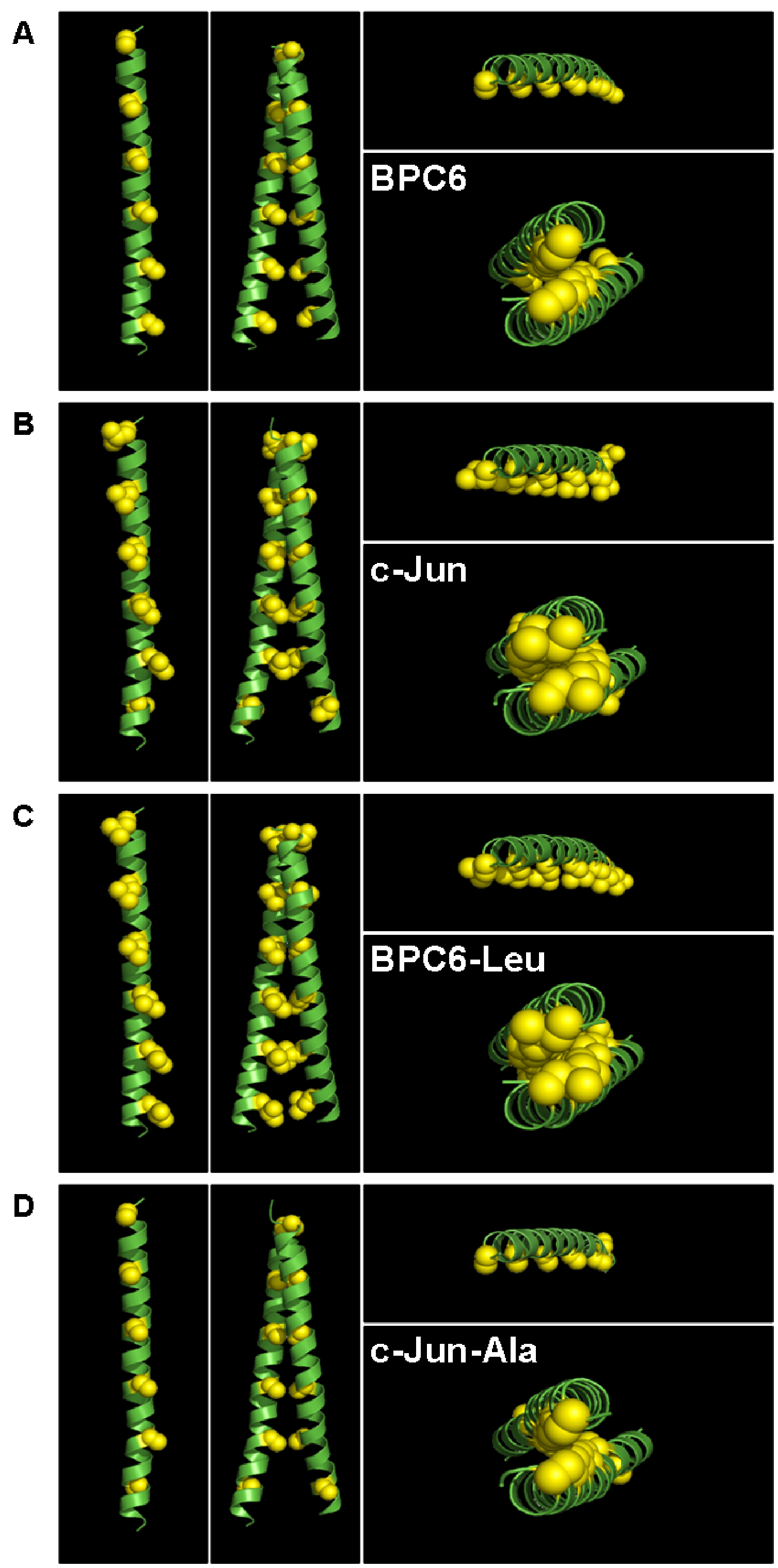

Supplement: Figure S7 — Homology models of monomeric and homodimeric coiled-coil structures. The homology models of the monomeric and homodimeric coiled-coil regions of BPC6, C-Jun, C-Jun-Ala and BPC6-Leu were computed by using the backbone coordinates of the C-Jun Leucine-zipper. Conserved alanine and leucine residues (both at ’d’ position of the register) in BPC6 and C-Jun or mutated alanines and leucins in BPC6-Leu or C-Jun-Ala were highlighted in yellow. The figure illustrates the identical models from two angles as monomers or homodimers. The monomeric molecules were fitted by using PyMOL (http://www.pymol.org), the dimers were subsequently computed and displayed using AMBER (http://ambermd.org/). (TIF) [file pone.0016070.s007.tif]

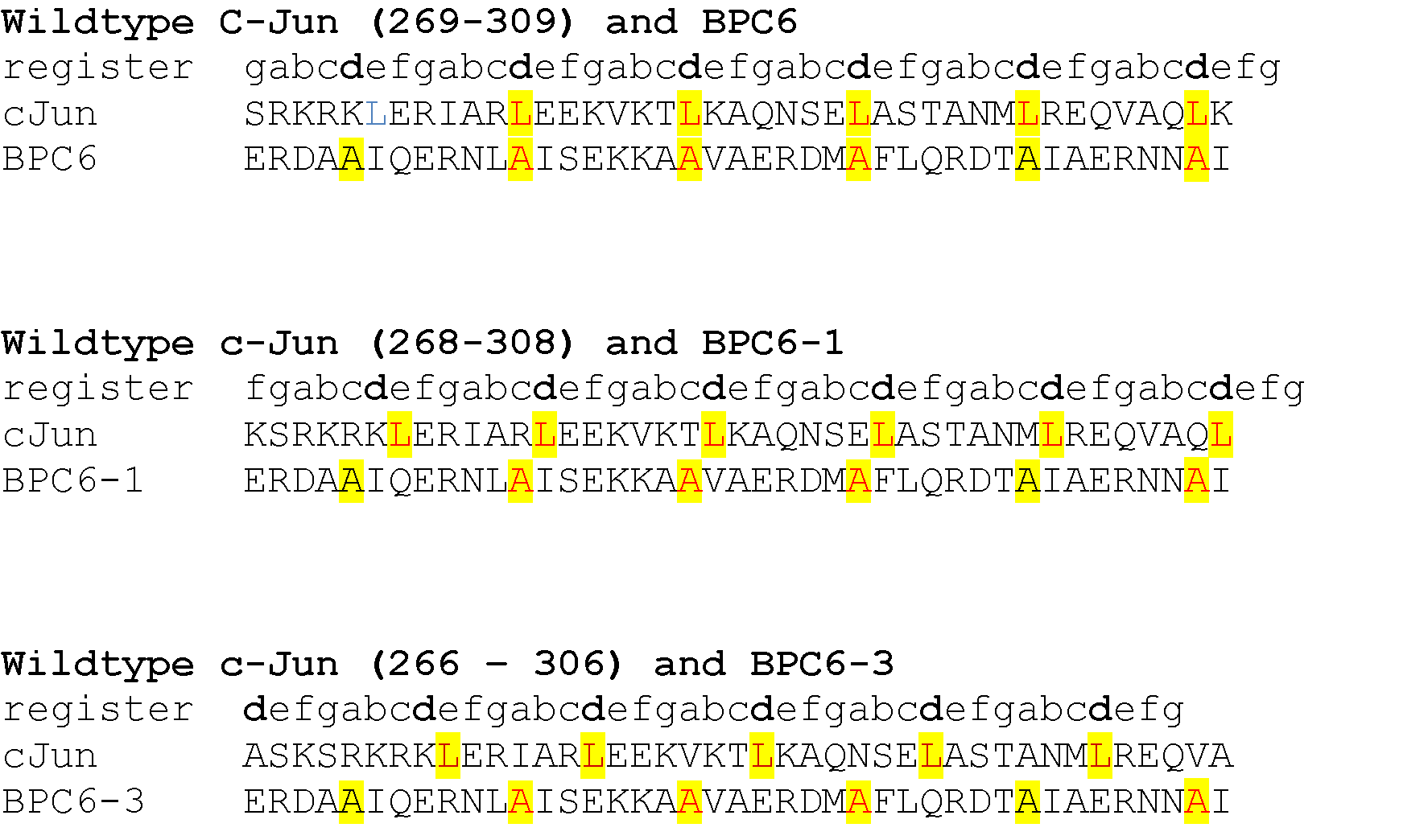

Supplement: Figure S8 — Alignment of the native AtBPC6 and the register shifted BPC versions with C-Jun. The register of the C-Jun alpha-helix is shown on top of each alignment. Conserved alanine or leucine residues are highlighted in red and yellow background. The native sequences are aligned to fit the conserved amino acids at ‘d’-position of the register. In BPC6-1 the register is shifted by one position, in BPC6-3 it is shifted by three positions, respectively. Thus, the conserved alanines are now at positions ‘c’ (BPC6-1) or ‘a’ (BPC6-3). (TIF) [file pone.0016070.s008.tif]

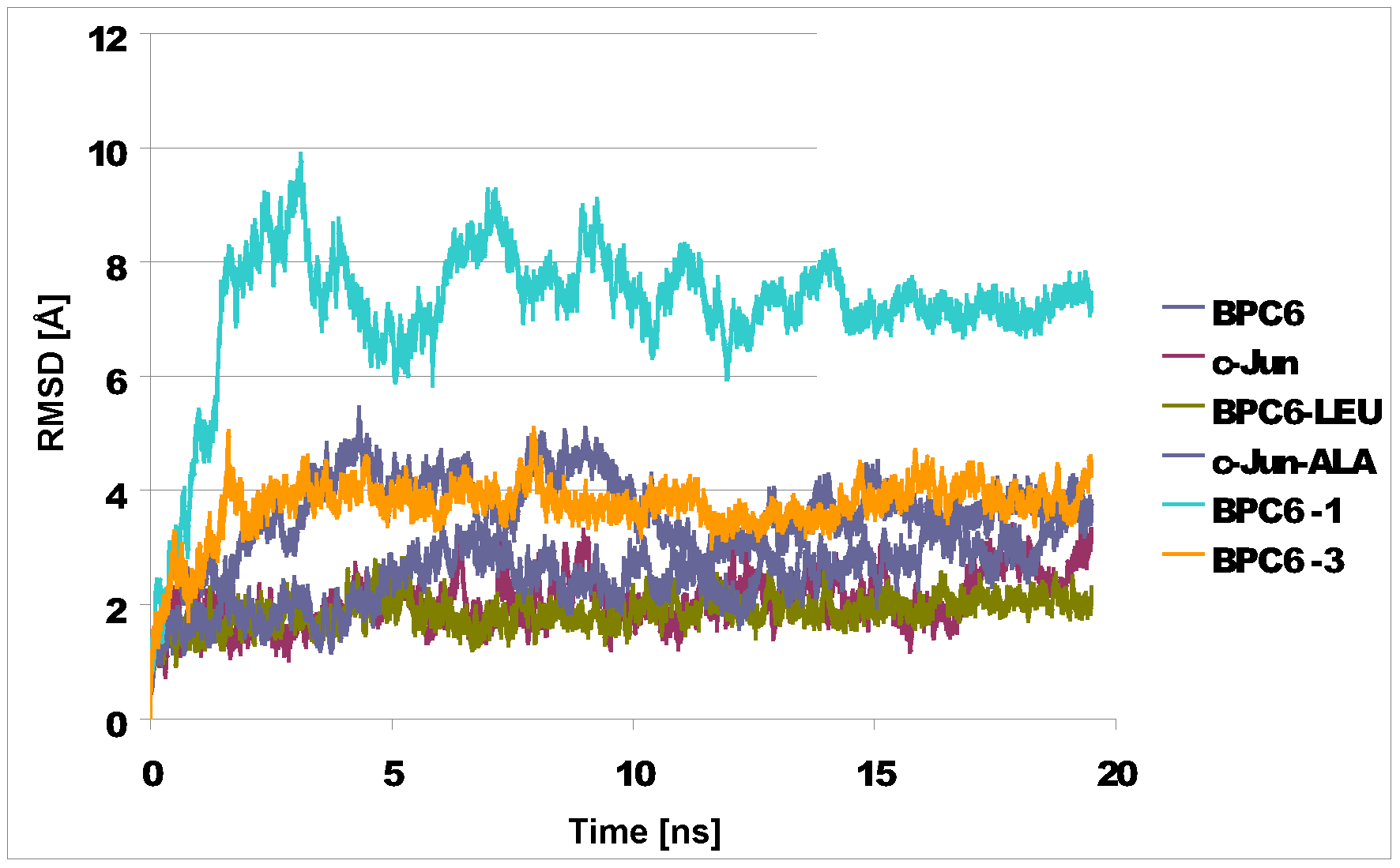

Supplement: Figure S9 — RMSD of the backbone atoms of the dimeric models during the production run (19.5ns). The 19.5ns production run was performed at constant pressure and constant temperature. Coordinates for analysis were saved every 1 ps. Root-mean-square deviations (RMSD) of the backbone atoms of the six indicated protein structures were computed from the MD trajectory relative to the initial structures. (TIF) [file pone.0016070.s009.tif]

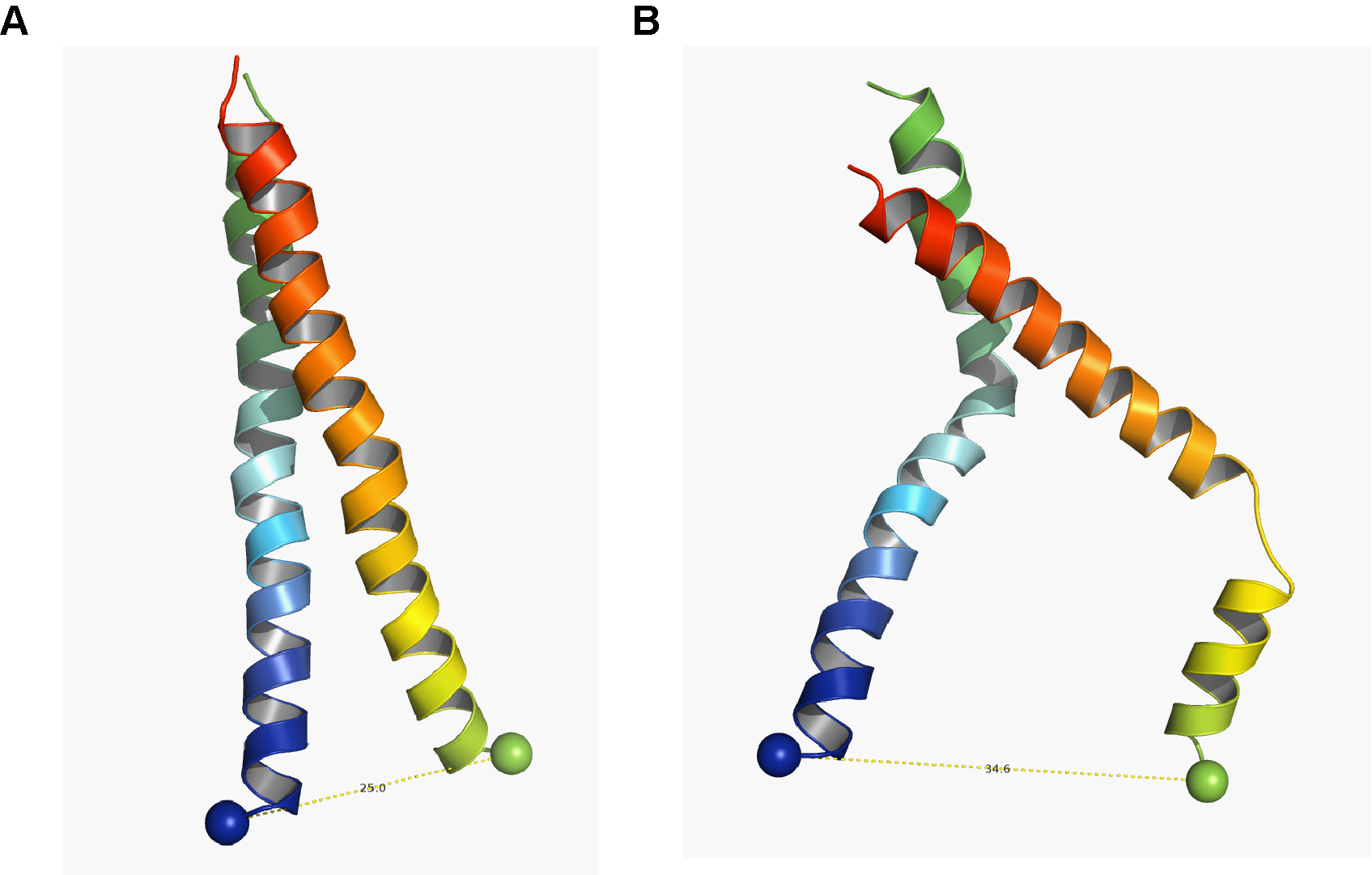

Supplement: Figure S10 — Homology model of the register shifted BPC6-1. The homology model of the coiled-coil structure of AtBPC6 was computed by using the backbone coordinates of the C-Jun Leucine-zipper, but shifted by −1 register. The conserved alanine residues in BPC6 at ‘d’-position are not at ‘c’-position. Note that within the short period of 2.3ns the two helices departed. (A) start position; (B) 2.3ns of equilibration. (TIF) [file pone.0016070.s010.tif]

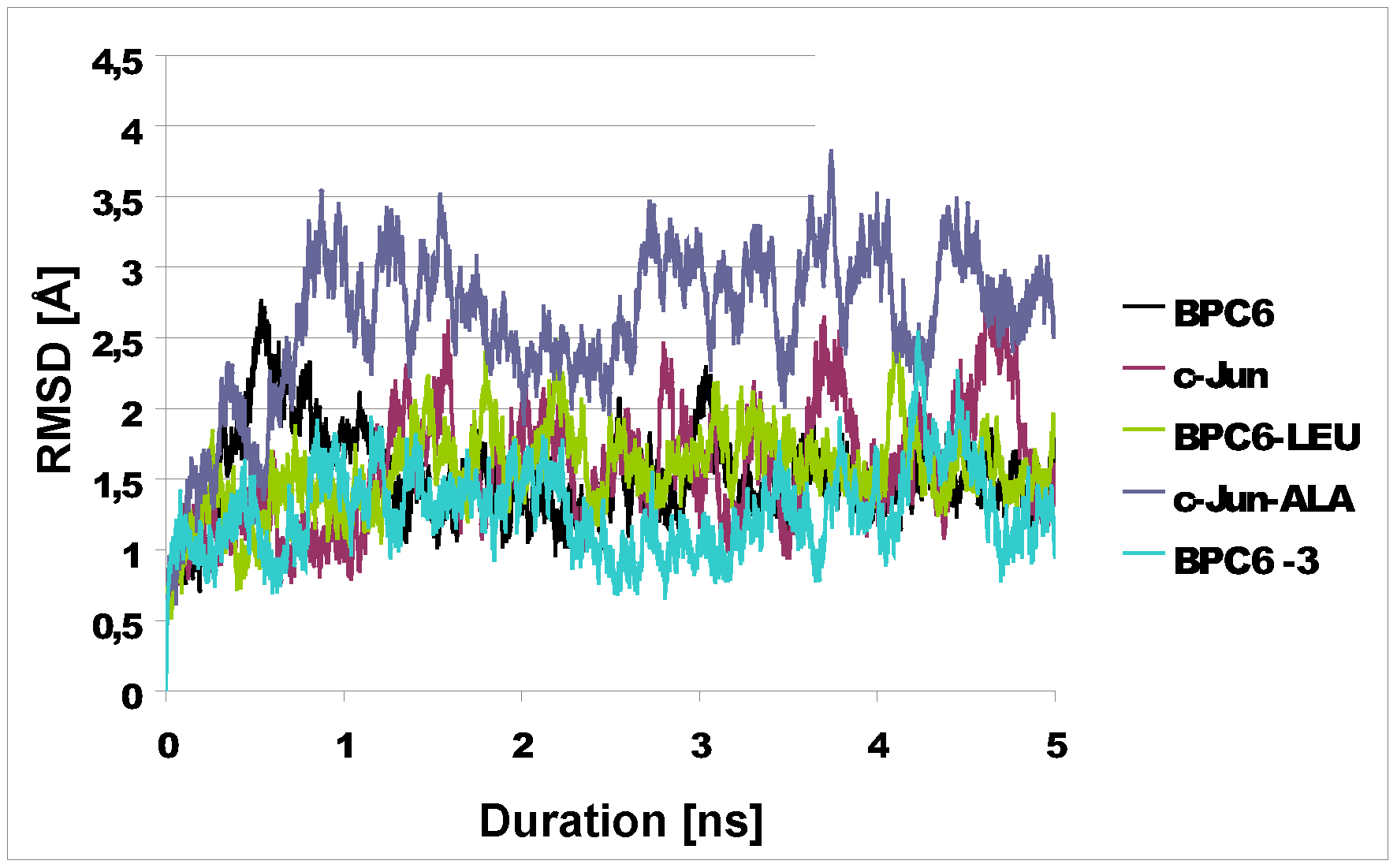

Supplement: Figure S11 — RMSD of the backbone atoms of the dimeric models during the time period that was used for the calculation of the binding free energies. Schematic overview of the 5 nanosecond periods from the RMSD of the backbone atoms that were taken for the calculation of ΔGBinding: BPC6 - 15.5ns to 19.5ns (black line); C-Jun - 11.0ns to 16.0ns (red line); BPC6-LEU - 15.5ns to 19.5ns (green line); c-Jun-ALA - 11.0ns tp 16.0ns (violett line); BPC6-3 - 15.5ns to 19.5ns (cyan). (TIF) [file pone.0016070.s011.tif]
